# Transmembrane Helices Are an Over-Presented and Evolutionarily Conserved Source of Major Histocompatibility Complex Class I and II Epitopes

**DOI:** 10.3389/fimmu.2021.763044

**Published:** 2022-01-11

**Authors:** Richèl J. C. Bilderbeek, Maksim V. Baranov, Geert van den Bogaart, Frans Bianchi

**Affiliations:** Department of Molecular Immunology, Groningen Biomolecular Sciences and Biotechnology (GBB) Institute, University of Groningen, Groningen, Netherlands

**Keywords:** antigen presentation, membrane proteins, adaptive immunity, transmembrane domain, epitopes, MHC-I, MHC-II, evolutionary conservation

## Abstract

Cytolytic T cell responses are predicted to be biased towards membrane proteins. The peptide-binding grooves of most alleles of histocompatibility complex class I (MHC-I) are relatively hydrophobic, therefore peptide fragments derived from human transmembrane helices (TMHs) are predicted to be presented more often as would be expected based on their abundance in the proteome. However, the physiological reason of why membrane proteins might be over-presented is unclear. In this study, we show that the predicted over-presentation of TMH-derived peptides is general, as it is predicted for bacteria and viruses and for both MHC-I and MHC-II, and confirmed by re-analysis of epitope databases. Moreover, we show that TMHs are evolutionarily more conserved, because single nucleotide polymorphisms (SNPs) are present relatively less frequently in TMH-coding chromosomal regions compared to regions coding for extracellular and cytoplasmic protein regions. Thus, our findings suggest that both cytolytic and helper T cells are more tuned to respond to membrane proteins, because these are evolutionary more conserved. We speculate that TMHs are less prone to mutations that enable pathogens to evade T cell responses.

## 1 Introduction

Our immune system fights diseases and infections from pathogens, such as fungi, bacteria or viruses. An important part of the acquired immune response, that develops specialized and more specific recognition of pathogens than the innate immune response, are T cells which recognize peptides, called epitopes, derived from antigenic proteins presented on Major Histocompatibility Complexes (MHC) class I and II on the cell surface.

The MHC proteins are heterodimeric complexes encoded by the HLA (Human Leukocyte Antigens) genes. In humans, the peptide binding groove of MHC-I is made by only the alpha subunit. There are three classical alleles of MHC-I, hallmarked by a highly polymorphic alpha chain called HLA-A, HLA-B and HLA-C, that all present epitopes to cytolytic T cells. For MHC-II, both the alpha and the beta chains contribute to the peptide binding groove. There are three classical alleles of MHC-II as well, called HLA-DR, HLA-DQ and HLA-DP, that all present epitopes to helper T cells. Each MHC complex can present a subset of all possible peptides. For example, HLA-A and HLA-B have no overlap in which epitopes they bind (1). Moreover, the HLA genes of humans are highly polymorphic, with hundreds to thousands of different alleles, and each different allele presents a different subset of peptides (2).

Humans express a limited set of MHC alleles and therefore an individual’s immune system detects only a fraction of all possible peptide fragments. However, at the population level, the coverage of pathogenic peptides that are detected is very high, because of the highly polymorphic MHC genes. It is therefore believed that MHC polymorphism improves immunity at the population level, as mutations in a protein that disrupt a particular MHC presentation at the individual level, so-called escape mutations, will not affect MHC presentation for all alleles present in the population (3).

Many studies are aimed at identifying the repertoire of epitopes that are presented in any of the different alleles to determine which epitopes will result in an immune response, as this will for instance aid the design of vaccines. These studies have led to the development of prediction algorithms that allow for very reliable *in silico* predictions of the peptide binding affinities (4–6). For example, S. Tang et al. (6) found that, of the 432 peptides that were predicted to bind to an MHC allele, 86% were experimentally confirmed to do so.

Using these prediction algorithms, we recently showed that peptides derived from transmembrane helices (TMHs) are likely to be more frequently presented by MHC-I than expected based on their abundance (7), which is in line with a previous study by Istrail et al. (8), demonstrating that N-terminal signal sequences are likely to be presented within major histocompatibility complexes, due their hydrophobic nature. Moreover, we showed that some well-known immunodominant peptides stem from TMHs. This over-presentation is attributed to the fact that the peptide-binding groove of most MHC-I alleles is relatively hydrophobic, and therefore hydrophobic TMH-derived peptides have a higher affinity to bind than their soluble hydrophilic counterparts.

TMHs are hydrophobic as they need to span the hydrophobic lipid bilayer of cellular membranes. They consist of an alpha helix of, on average, 23 amino acids in length. TMHs can also be predicted with high accuracy from a protein sequence by bioinformatics approaches (9–14). For example, a study by Jones (12) found that, from 184 transmembrane proteins (TMPs) with known topology, 80% of the TMH predictions of these proteins matched the experimental findings. TMHs are common structures in the proteins of humans and microbes. Different TMH prediction tools estimate that 15-39% of all proteins in the human proteome contain at least one TMH (15). However, the physiological reason why peptides derived from TMHs would be presented more often than peptides stemming from soluble (i.e., extracellular or cytoplasmic) protein regions is unknown. In this study, we hypothesized that the presentation of TMH residues is evolutionarily preferred, since TMHs are less prone to undergo escape mutations. One reason to expect such a reduced variability (and hence evolutionary conservation) in TMHs, is that these are restricted in their variability by the functional requirement to span a lipid bilayer. This limits many of the amino acids present in TMHs to have hydrophobic side chains (16, 17). Therefore, we speculated that the TMHs of pathogens might have a lower chance to develop escape mutations, as that will result in a dysfunctional TMH and render the protein inactive.

This study had two objectives. First, we aimed to generalize our findings by predicting the antigenic presentation from different kingdoms of life in both MHC-I and -II. From these *in silico* predictions, we conclude that TMH-derived epitopes from a human, viral and bacterial proteome are likely to be presented more often than expected by chance for most alleles of MHC-I and II. We confirmed the presentation of TMH-derived peptides by re-analysis of peptides from The Immune Epitope Database (IEDB) (18). Second, we tested our hypothesis that TMHs are more evolutionary conserved than soluble protein regions. Our analysis of human single nucleotide polymorphisms (SNPs) showed that random point mutations are indeed less likely to occur within TMHs. These findings strengthen the emerging notion that TMHs are important for the T cell-mediated adaptive immune system, and hence are of overlooked importance in vaccine development.

## 2 Methods

### 2.1 Predicting TMH Epitopes

To predict how frequently epitopes overlapping with TMHs are presented, a similar analysis strategy was applied as described in (7) for several alleles of both MHC-I and MHC-II, and for a human, viral and bacterial proteome. To summarize, for each proteome, all possible 9-mers (for MHC-I) or 14-mers (MHC-II) were derived. For each of these peptides, we determined if it overlapped with a predicted TMH and if it was predicted to bind to the most frequent alleles of each MHC allele.

For MHC-I, 9-mers were used, as this is the length most frequently presented in MHC-I and was used in our earlier study (7). For MHC-II, 14-mers were used, as this is the most frequently occurring epitope length (19). A human (UniProt ID UP000005640_9606), viral (SARS-CoV-2, UniProt ID UP000464024) and bacterial (*Mycobacterium tuberculosis*, UniProt ID UP000001584) reference proteome was used. TMHMM (9) was used to predict the topology of the proteins within these proteomes. To predict the affinity of an epitope to a certain HLA allele, EpitopePrediction (7) for MHC-I and MHCnuggets (20) for MHC-II was used. Both MCH-I and MHC-II alleles were selected to have a high prevalence in the population, where the alleles of MHC-I are the alleles representing the 13 supertypes with over 99.6% coverage of the population’s MHC-I repertoire as defined by (1, 21), and the 21 MHC-II alleles, have a phenotypic frequency of 14% or more in the human population (22).

We define a protein to be a binder if, for a certain MHC allele, any of its 9-mer or 14-mer peptides have an IC50 value in the lowest 2% of all peptides within a *proteome* (**Supplementary Tables S1**, **S2**), this differs from our previous study where we defined a binder as having an IC50 in the lowest 2% of the peptides within a *protein*. This revised definition precludes bias of proteins that give rise to no or only very few MHC epitopes. To verify that the slight change in method yields similar results, a side by side comparison is shown in the **Supplementary Materials**, **Supplementary Figures S1**, **S2**.

### 2.2 TMH Epitopes Obtained From Experimental Data

To obtain experimental confirmation that peptides stemming from TMHs are presented by MHC-I and MHC-II, we mined the IEDB (18) for confirmed human MHC-ligands. We queried the IEDB for all linear epitopes obtained from MHC ligand assays in healthy humans, carrying the MHC alleles as used in this study. From these epitopes, we kept those that were present exactly once in the human reference proteome with UniProt ID UP000005640_9606. We concluded that the epitope overlapped with a TMH if at least 1 amino acid was overlapping with a TMH, as predicted with TMHMM (9), from which we concluded if the epitope is overlapping with a TMH with at least 1 amino acid.

The full analysis can be found at (https://github.com/richelbilderbeek/bbbq_article_issue_157).

#### 2.2.1 Evolutionary Conservation of TMHs

To determine the evolutionary conservation of TMHs, we first collected human single nucleotide polymorphisms (SNPs) resulting in a single amino acid substitution and determined if this occurred within a predicted TMH or not.

As a data source, multiple NCBI (https://www.ncbi.nlm.nih.gov/) databases were used: the *dbSNP* (23) database, which contains 650 million cataloged non-redundant human variations (called *RefSNPs*, https://www.ncbi.nlm.nih.gov/snp/docs/RefSNP_about/), and the databases *gene* [for gene names (24)] and *protein* [for proteins sequences (25)].

The first query was a call to the *gene* database for the term ‘membrane protein’ (in all fields) for the organism *Homo sapiens*. This resulted in 1,077 gene IDs (on December 2020). The next query was a call to the *gene* database to obtain the gene names from the gene IDs. Per gene name, the *dbSNP* NCBI database was queried for variations associated with the gene name. As the NCBI API constrains its users to three calls per second (to assure fair use), we had to limit the extent of our analysis.

The number of SNPs was limited to the first 250 variations per gene, resulting in ≈ 61k variations. Only variations that result in a SNP for a single amino acid substitution were analyzed, resulting in ≈ 38k SNPs. The exact amounts can be found in the supplementary materials, **Supplementary Tables S3**, **S4**.

SNPs were picked based on ID number, which is linked to their discovery date. To verify that these ID numbers are unrelated to SNP positions, the relative positions of all analyzed SNPs in a protein were determined. This analysis showed no positional bias of the SNPs, as shown in **Supplementary Figure S3**.

Per SNP, the *protein* NCBI database was queried for the protein sequence. For each protein sequence, the protein topology was determined using PureseqTM. Using these predicted protein topologies, the SNPs were scored to be located within or outside TMHs.

## 3 Results

### 3.1 TMH-Derived Peptides Are Predicted to Be Over-Presented in MHC-I

**Figure 1:**
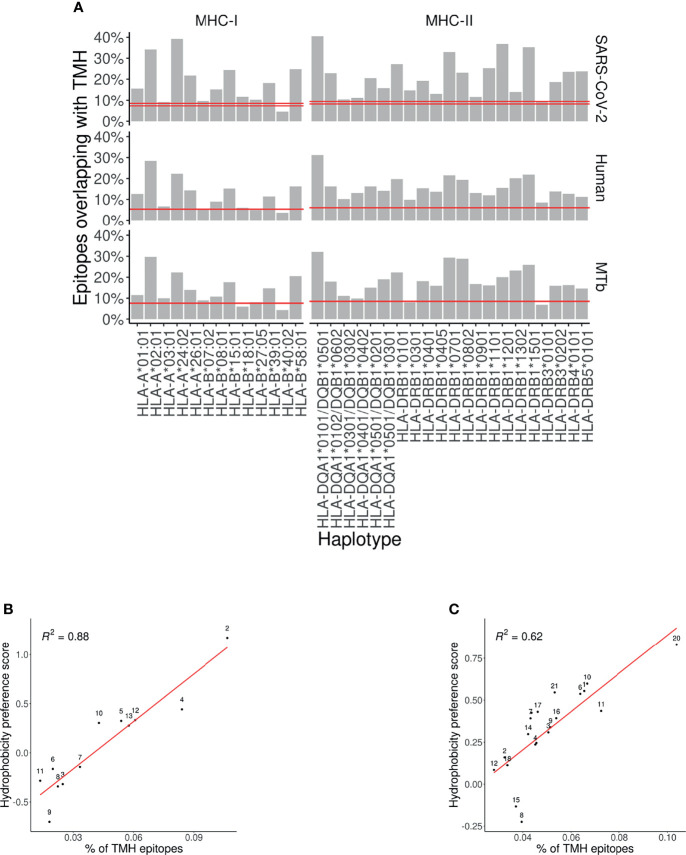
Over-presentation of TMH-derived epitopes on most MHC-I and -II alleles **(A)** The percentage of epitopes for MHC-I and -II alleles that are predicted to overlap with TMHs for the proteomes of SARS-CoV-2 (top row), human (middle row) and *M. tuberculosis* (MtB; bottom row). The pair of horizontal red lines in each plot indicate the lower and upper bound of the 99% confidence interval. See **Supplementary Tables S5** and **S7** for the exact TMH and epitope counts. **(B, C)** Correlation between the percentages of predicted TMH-derived epitopes and the hydrophobicity score of all predicted epitopes for human MHC-I **(B)** and MHC-II alleles **(C)**. Diagonal red line: linear regression analysis. Labels are shorthand for the HLA alleles, see the **Supplementary Table S8** for the names.

**Figure 1A** shows the predicted presentation of TMH-derived peptides in MHC-I, for a human, viral and bacterial proteome. Per MHC-I allele, it shows the percentage of binders that overlap with a TMH with at least one residue. The horizontal line shows the expected percentage of TMH-derived epitopes that would be presented, if TMH-derived epitopes would be presented just as likely as epitopes derived from soluble regions, when assuming equal incidence of soluble and TMH-derived epitope presentation. For 11 out of 13 MHC-I alleles, TMH-derived epitopes are predicted to be presented more often than the null expectation, for a human and bacterial proteome. For the viral proteome, 12 out of 13 MHC-I alleles present TMH-derived epitopes more often than expected by chance. The extent of the over-presentation between the different alleles is similar for the probed proteomes, which strengthens our previous conclusion (7) that the hydrophobicity of the MHC-binding groove is the main factor responsible for the predicted over-presentation of TMH-derived peptides.

### 3.2 TMH-Derived Peptides Are Predicted to Be Over-Presented in MHC-II

We next wondered if the over-representation of TMH-derived peptides would also be present for MHC-II. **Figure 1A** shows the percentages of MHC-II epitopes predicted to be overlapping with TMHs for our human, viral and bacterial proteomes. We found that TMH-derived peptides are over-presented in all of the 21 MHC-II alleles, for a human, bacterial and viral proteome, except for HLA-DRB3*0101 in *M. tuberculosis*. See **Supplementary Table S5** for the exact TMH and epitope counts.

### 3.3 The Over-Presentation of TMH-Derived Peptides Is Caused by the Hydrophobicity of the MHC Peptide Binding Groove

For MHC-I, we previously showed that the over-presentation of TMH-derived peptides is caused by the hydrophobicity of the peptide binding grooves (7). **Figures 1B**, **C** show the extent of over-presentation of TMH-derived epitopes as a function of the hydrophobicity preference score for the different human MHC alleles. An assumed linear correlation explains 88% of the variability in MHC-I. For MHC-II, 62% of the variability is explained by hydrophobicity. This indicates that TMH-derived peptides are over-presented, because the peptide binding grooves of most MHC-I and -II alleles are relatively hydrophobic.

### 3.4 Experimental Validation of Presentation of TMH-Derived Peptides

The Immune Epitope Database (IEDB) from the National Institutes of Health contains millions of linear epitope sequences obtained by MHC ligand assays. For the MHC alleles used in this study, we obtained 54,303 and 2,484 linear epitope sequences for the MHC-I and MHC-II alleles from human origin respectively. There are relatively few epitopes for MHC-II, as MHC-II has many more different alleles than MHC-I, whereas we selected only the human epitopes found for the 21 MHC-II alleles used in this study.

**Figure 2:**
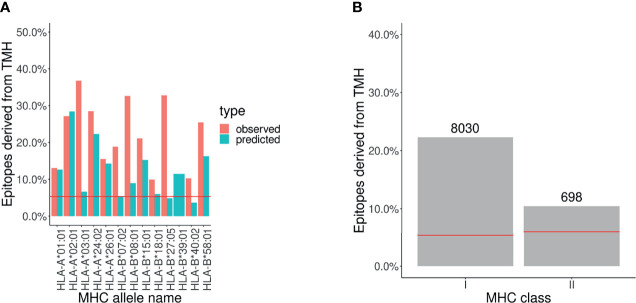
Analysis of epitope database shows that TMH derived epitopes are over-presented. The percentage of epitopes for MHC-I and -II alleles that overlap with TMHs that are presented. The pair of horizontal red lines in each plot indicate the lower and upper bound of the 99% confidence interval. Note that only one line is visible as this interval is relatively narrow. Alleles are listed in **Supplementary Table S8**. **(A)** Observed and predicted percentage of TMH-derived epitopes for MHC-I alleles. **(B)** MHC ligands from IEDB corresponding to TMH-derived epitopes. The numbers above the bars denotes the number of TMH derived epitopes obtained.

**Figure 2A** and **Supplementary Figure S4** show there are similar levels of over-presentation of TMH-derived epitopes between (1) the percentage of TMH-derived epitopes that is reported in the IEDB database versus (2) the percentage of TMH-derived epitopes that is predicted to be presented in MHC-I alleles. For MHC-II alleles, there were too few epitopes per MHC allele to result in an informative figure.

In **Figure 2B** we grouped all the epitopes presented by MHC-I and MHC-II alleles by the percentage of TMH-derived epitopes, which are 22% and 10%, respectively.

These findings robustly confirm that epitopes derived from human TMHs are presented in both MHC-I and MHC-II, and support that they are over-presented. See the **Supplementary Table S6** for the exact values.

We also mined the IEDB database for epitopes for any type of T cell response from the specified alleles. From the total reports, 36% and 7% concerned TMH-derived epitopes in MHC class I and II, respectively (see **Supplementary Figure S5**).

This data confirms that not only TMH derived epitopes are presented on MHC, but this also elicits T cell mediated immune responses.

### 3.5 Human TMHs Are Evolutionarily Conserved

We addressed the question whether there is an evolutionary advantage in presenting TMHs. We determined the conservation of TMHs by comparing the occurrences of SNPs located in TMHs or soluble protein regions for the genes coding for membrane proteins. We obtained 911 unique gene names associated with the phrase ‘membrane protein’, which are genes coding for both membrane-associated proteins (MAPs, which have no TMH) and transmembrane proteins (TMPs, which have at least one TMH). These genes are linked to 4,780 protein isoforms, of which 2,553 are predicted to be TMPs and 2,237 proteins are predicted to be MAPs. We obtained 37,630 unique variations, of which 9,621 are SNPs that resulted in a straightforward amino acids substitution, of which 6,062 were located in predicted TMPs. See supplementary **Supplementary Table S3** and **S4** for the detailed numbers and distributions of SNPs.

**Figure 3:**
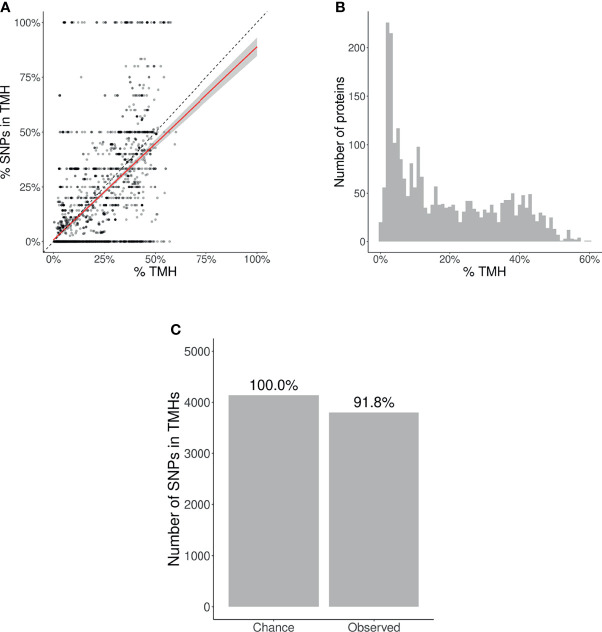
Evolutionary conservation of human TMHs. **(A)** Percentage of SNPs found in TMHs. Each point shows for one protein the predicted percentage of amino acids that are part of a TMH (*x*-axis) and the observed occurrence of SNPs being located within a TMH (*y*-axis). The dashed diagonal line shows the line of equality (i.e., equal conservation of TMHs and soluble protein regions). The diagonal red line indicates a linear fit, the gray area its 95% confidence interval. **(B)** Distribution of the percentages of TMH in the TMPs used in this study. **(C)** The number of SNPs in TMHs as expected by chance (left bar) and found in the dbSNP database (right bar). Percentages show the relative conservation of SNPs in TMHs found relative to stochastic chance.

Per protein, we calculated two percentages: (1) the percentage of a protein sequence length bearing TMHs, and (2) the percentage of SNPs located within these predicted TMHs. Each percentage pair was plotted in **Figure 3A**. The proportion of SNPs found in TMHs varied from none (i.e., all SNPs were in soluble regions) to all (i.e., all SNPs were in TMHs). To determine if SNPs were randomly distributed over the protein, we performed a linear regression analysis, and added a 95% confidence interval on this regression. This linear fit nearly goes through the origin and has a slope below the line of equality, which shows that less SNPs are found in TMHs than expected by chance.

We determined the probability to find the observed amount of SNPs in TMHs by chance, i.e., when assuming SNPs occur just as likely in soluble domains as in TMHs. We used a binomial Poisson distribution, where the number of trails (*n*) equals the number of SNPs, which is 21,208. The probability of success for the *i*th TMP (*p*_*i*), is the percentage of residues within a TMH per TMP. These percentages are shown as a histogram in **Figure 3B**. The expected number of SNPs expected to be found in TMHs by chance equals Σ*p* ≈ 4,141. As we observed 3,803 SNPs in TMHs, we calculated the probability of having that amount or less successes. We used the type I error cut-off value of *α* = 2.5%. The chance to find, within TMHs, this amount or less SNPs equals 6.8208·10^–11^. We determined the relevance of this finding, by calculating how much less SNPs are found in TMHs, when compared to soluble regions, which is the ratio between the number of SNPs found in TMHs versus the number of SNPs as expected by chance. In effect, per 1000 SNPs found in soluble protein domains, one finds 918 SNPs in TMHs, as depicted (as percentages) in **Figure 3C**.

**Figure 4:**
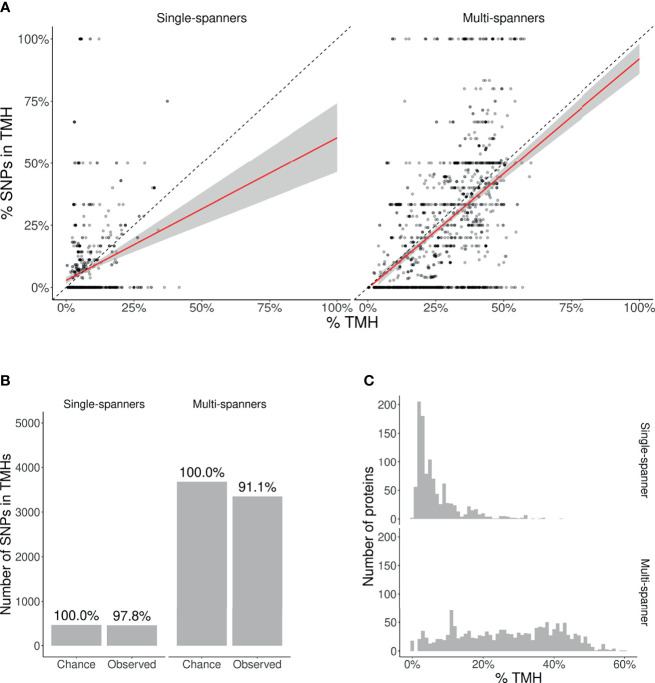
| Membrane proteins with multiple TMHs are evolutionary more conserved than proteins with only a single TMH. **(A)** Percentage of SNPs found in TMPs predicted to have only a single (left) or multiple (right) TMHs. Each point shows for one protein the predicted percentage of amino acids that are part of a TMH (*x*-axis) and the observed occurrence of SNPs being located within a TMH (*y*-axis). The dashed diagonal lines show the line of equality (i.e., equal conservation of TMHs and soluble protein regions). The diagonal red lines indicate a linear fit, the gray areas their 95% confidence intervals. **(B)** The number of SNPs in TMHs as expected by chance and observed in the dbSNP database, for TMPs with one TMH (single-spanners) and multiple TMHs (multi-spanners). Percentages show the relative conservation of SNPs in TMHs found relative to the stochastic chances. **(C)** Distribution of the proportion of amino acids residing in the plasma membrane.

We split this analysis for TMPs containing only a single TMH (so-called single-membrane spanners) and TMPs containing multiple TMHs (multi-membrane spanners). We hypothesized that single-membrane spanners are less conserved than multi-membrane spanners, because multi-membrane spanners might have protein-protein interactions between their TMHs, for example to accommodate active sites, and thus might have additional structural constraints. From the split data, we did the same analysis as for the total TMPs. **Figure 4A** shows the percentages of TMHs for individual proteins as a function of the percentage of SNPs located in TMHs. For both single- and multi-spanners, a linear regression shows that less SNPs are found in TMHs, than expected by chance.

We also determined the probability to find the observed amount of SNPs by chance in single- and multi-spanners. For single-spanners, we found 452 SNPs in TMH, where ≈ 462 were expected by chance. The chance to observe this or a lower number by chance is 0.319. As this chance was higher than our *α* = 0.025, we consider this no significant effect. For the multi-spanners, we found 3,351 SNPs in TMH, where ≈ 3, 678 were expected by chance. The chance to observe this or a lower number by chance is 8.31584·10^–12^, which means this number is significantly less as explained by variation. The TMHs of multi-spanners are thus significantly more conserved than soluble protein regions, whereas this is not the case for single-spanners.

Also, for single- and multi-spanners, we determined the relevance of this finding by calculating where and how much less SNPs are found in TMHs when compared to soluble regions, as depicted in **Figures 4B, C**. In effect, per 1,000 SNPs found in soluble protein domains, one finds 978 SNPs in TMHs of single-spanners and 911 SNPs in TMHs of multi-spanners.

## 4 Discussion

Epitope prediction is important to understand the immune system function and for the design of vaccines. In this study, we provide evidence that epitopes derived from TMHs are a major source of MHC epitopes. Our bioinformatics predictions indicate that the TMH-derived epitope repertoire is larger than expected by chance for both MHC-I and MHC-II, regardless of the organism. Moreover, reanalysis of MHC-ligands from the IEDB database confirmed the presentation of TMH-derived epitopes. Therefore, it seems likely that TMH-derived epitopes would also result in enhanced T cell responses, although the conservation of TMHs might promote the deletion of T cells responsive to TMH-derived epitopes by central tolerance mechanisms. Finally, our SNP analysis shows that TMHs are evolutionary more conserved than solvent-exposed protein regions.

### 4.1 Mechanism of MHC Presentation of TMH-Derived Epitopes

Although our data show that TMH-derived epitopes are presented in all classical MHC-I and MHC-II alleles, the molecular mechanisms of how integral membrane proteins are processed for MHC presentation are largely unknown (7). Most prominently, the fundamental principles of how TMHs are extracted from their hydrophobic lipid environments into the aqueous vacuolar lumen, leading to subsequent proteolytic processing are unresolved.

A first possibility is that the extraction of TMPs from the membrane is mediated by the ER-associated degradation (ERAD) machinery. For MHC class I (MHC-I) antigen presentation of soluble proteins, the loading of the epitope primarily occurs at the endoplasmatic reticulum (ER). The chaperones tapasin (TAPBP), ERp57 (PDIA3), and calreticulin (CALR) (26) first assemble and stabilize the heavy and light chains of MHC-I. Later, this complex binds to the transporter associated with antigen processing (TAP) leading to the formation of the so-called peptide-loading complex (PLC). The PLC drives import of peptides into the ER and mediates their subsequent loading into the peptide-binding groove of MHC-I (27). Membrane proteins first will have to be extracted from the membrane before they become amenable to this MHC-I loading by the PLC. In the ER, this process can be orchestrated by the ERAD machinery, consisting of several chaperones that recognize TMPs, ubiquitinate them, and extract them from the ER membrane into the cytosol (retrotranslocation) for proteasomal degradation (28, 29). Similar to the peptides generated from soluble proteins, the TMP-derived peptides might then be re-imported by TAP into the ER for MHC-I loading. This ERAD-driven antigen retrotranslocation might be facilitated by lipid bodies (LBs) (30), since LBs can serve as cytosolic sites for ubiquitination of ER-derived cargo (31).

A second possibility is that TMPs are proteolytically processed by intramembrane proteases that cleave TMHs while they are still membrane embedded. Supporting this hypothesis is the well-established notion that peptides generated by signal peptide peptidases (SPPs), an important class of intramembrane proteases that cleave TMH-like signal sequences, are presented on a specialized class of MHC-I called HLA-E (32). The loading of peptides generated by SPP onto MHC-I does not depend on the proteosome and TAP, possibly because the peptides are directly released into the lumen of the ER (32). However, this mechanism cannot explain how most membrane proteins can be processed for antigen presentation, because SPPs only cleave TMH-like signal sequences at their C-termini, and N-terminal domains will hence not be removed. Nevertheless, the presentation of peptides with a high hydrophobicity index was shown to be independent of TAP as well (33), suggesting that the TMH peptides might perhaps be released directly in the ER lumen by other intramembrane proteases.

A third possibility is that peptide processing and MHC-loading occur in multivesicular bodies (MVBs) (32). TMPs can be routed from the plasma membrane and other organelles by vesicular trafficking to endosomes. Eventually, these TMPs can be sorted by the endosomal sorting complexes required for transport (ESCRT) pathway into luminal invaginations that pinch off from the limiting membrane and form intraluminal vesicles. This thus results in MVBs where the membrane proteins destined for degradation are located in intraluminal vesicles. Upon the fusion of MVBs with lysosomes, the entire intraluminal vesicles including the TMPs are degraded (34). *Via* this mechanism, TMPs might well be processed for antigen presentation, particularly since the loading of MHC-II molecules is well understood to occur in MVBs (35–37). However, such processing of membrane proteins in MVBs for antigen presentation poses a problem, because complexes of HLA-DR with its antigen-loading chaperon HLA-DM were only observed on intraluminal vesicles, but not on the limiting membranes of MVBs (37), indicating that epitope loading of MHC-II also occurs at intraluminal vesicles. This observation hence raises the question how the intraluminal vesicles carrying the TMPs destined for antigen presentation can be selectively degraded, while the intraluminal vesicles carrying the MHC-II remain intact. A second problem is that phagosomes carrying internalized microbes lack intraluminal vesicles, and it is hence unclear how TMPs from these microbes would be routed to MVBs for MHC-II loading (37).

Alternatively to the enzymatic degradation of lipids in MVBs by lipases (38, 39), they might be oxidatively degraded by reactions with radical oxygen species produced by the NADPH oxidase NOX2 (40). This oxidation can result in a destabilization and disruption of membranes (40) and might thereby lead to the extraction of TMPs. Due to the hydrophobic nature of TMHs, however, the extracted proteins will likely aggregate and it is unclear how these aggregates would be processed further for MHC loading.

### 4.2 Evolutionary Conservation of TMHs

In general, one might expect that evolutionary selection shapes an immune system where surveillance is directed towards protein regions essential for the survival, proliferation and/or virulence or pathogenic microbes, as these will be most conserved. In SARS-CoV-2, for example, there is preliminary evidence that the strongest selection pressure is directed upon residues that change its virulence (41). These regions, however, may only account for a small part of a pathogen’s proteome. Additionally, the structure and function of these essential regions might differ widely between different pathogenic proteins. Because of this scarcity and variance in targets, one can imagine that it will be mostly unfeasible to provide innate immune responses against such rare essential protein regions, as suggested in a study on influenza (42), where it was found that the selection pressure exerted by the immune system was either weak or absent.

Evolutionary selection of pathogens by a host’s immune system, however, is more likely to occur for protein patterns that are general, over patterns that are rare. While essential catalytic sites in a pathogenic proteome might be relatively rare, TMHs are common and thus might be a more feasible target for evolution to respond to. Indeed, we have found the signature of evolution when both factors, that is, TMHs and catalytic sites are likely to co-occur, which is in TMPs that span the membrane at least twice. In contrast to single-spanners, where we found no significant evolutionary conservation, the TMHs of multi-spanners are more evolutionary conserved than soluble protein regions. Likely, the TMHs in many multi-spanners need to interact which each other for correct protein structure and function and they might hence be more structurally constrained compared to the TMHs of single-spanners. Thus, we speculate that the human immune system is more attentive towards TMHs in multi-spanners, as these are evolutionarily more conserved.

There have been more efforts to assess the conservation of TMHs, using different methodologies. One such example is a study by Stevens and Arkin (43), in which aligned protein sequence data was used. Also this study found that TMHs are evolutionarily more conserved, as the mean amino acid substitution rate in TMHs is about ten percent lower, which is a similar value as we found. Another example is a study by Oberai, et al. (44) that estimated the conservation scores for TMHs and soluble regions based on alignments of evolutionary related proteins, and also found that TMHs are more conserved, with a conservation score that was 17% higher in TMHs. Note that the last study also found that mutations in human TMHs are likelier to cause a disease, in line with our conclusion that TMHs are more conserved.

Together, from this study, two important conclusions can be drawn. First, the MHC over-presentation of TMHs is likely a general feature and predicted to occur for most alleles of both MHC-I and -II and for humans as well as bacterial and viral pathogens. Second, TMHs are genuinely more evolutionary conserved than soluble protein motifs, at least in the human proteome.

## Data Availability Statement

Publicly available datasets were analyzed in this study. This data can be found here: https://www.ncbi.nlm.nih.gov/ and https://www.ncbi.nlm.nih.gov/snp/docs/RefSNP_about/. The data presented in the study are deposited in the Zenodo repository, accession number 10.5281/zenodo.5809139. All code, intermediate and final results are archived at https://github.com/richelbilderbeek/bbbq_article.

## Author Conributions

RB and FB conceived the idea for this research. MB helped with the proteome analysis of *M. tuberculosis*. RB wrote the code. RB, MB, GB, and FB wrote the article. All authors contributed to the article and approved the submitted version.

## Funding

FB is funded by a Veni grant from the Netherlands Organization for Scientific Research (016.Veni.192.026) and an Off-Road Grant from the Dutch Medical Science Foundation (ZonMW 04510011910005). GB is funded by a Young Investigator Grant from the Human Frontier Science Program (HFSP; RGY0080/2018), and a Vidi grant from the Netherlands Organization for Scientific Research (NWO-ALW VIDI 864.14.001). GB has received funding from the European Research Council (ERC) under the European Union’s Horizon 2020 research and innovation programme (grant agreement No. 862137).

## Conflict of Interest

The authors declare that the research was conducted in the absence of any commercial or financial relationships that could be construed as a potential conflict of interest.

## Publisher’s Note

All claims expressed in this article are solely those of the authors and do not necessarily represent those of their affiliated organizations, or those of the publisher, the editors and the reviewers. Any product that may be evaluated in this article, or claim that may be made by its manufacturer, is not guaranteed or endorsed by the publisher.
