# Supplementary material for: Transmembrane Helices Are an Over-Presented and Evolutionarily Conserved Source of Major Histocompatibility Complex Class I and II Epitopes
Source: Front Immunol. 2022 Jan 11;12:763044. doi: 10.3389/fimmu.2021.763044 (PMC8787072; doi:10.3389/fimmu.2021.763044)
Supplement: Supplementary file 1 [file DataSheet_1.pdf]

Supplementary Material

1 DIFFERENCES WITH BIANCHI ET AL., 2017

A part of this study does the same analysis as Bianchi et al., 2017. mainly concern the use of different software and a different definition of what an MHC binder is.

The earlier study defined a peptide an MHC binder if *within the protein* in which it was found, is was among the peptides with the 2% lowest IC50 values. This can be seen at `https://github.com/richelbilderbeek/bianchi_et_al_2017/blob/master/predict-binders.R`, where the binders are written to file.

However, in this study, an MHC binder is defined as a peptide within a *proteome* in which it is found, that is among the peptides with the 2% lowest IC50 values. Subsection 2 shows the IC50 values for a binder per MHC allele.

Our previous study used the TMHMM web server to predict TMHs. The desktop version of TMHMM, however, gives an error message on the 25 selenoproteins found in the human reference proteome. For the sake of reproducible research, we used the desktop version (as we can call it from scripts) and, due to this, we removed the selenoproteins from this analysis.

To verify if the previous and the current method give rise to notable difference, we show a side-by-side comparison in figures S1 and S2. The figures that MHC molecules that over-present or under-present TMH-derived epitopes, do so in both studies. The extent to which TMH-derived epitopes are presented, however, is more extreme in our current setup.

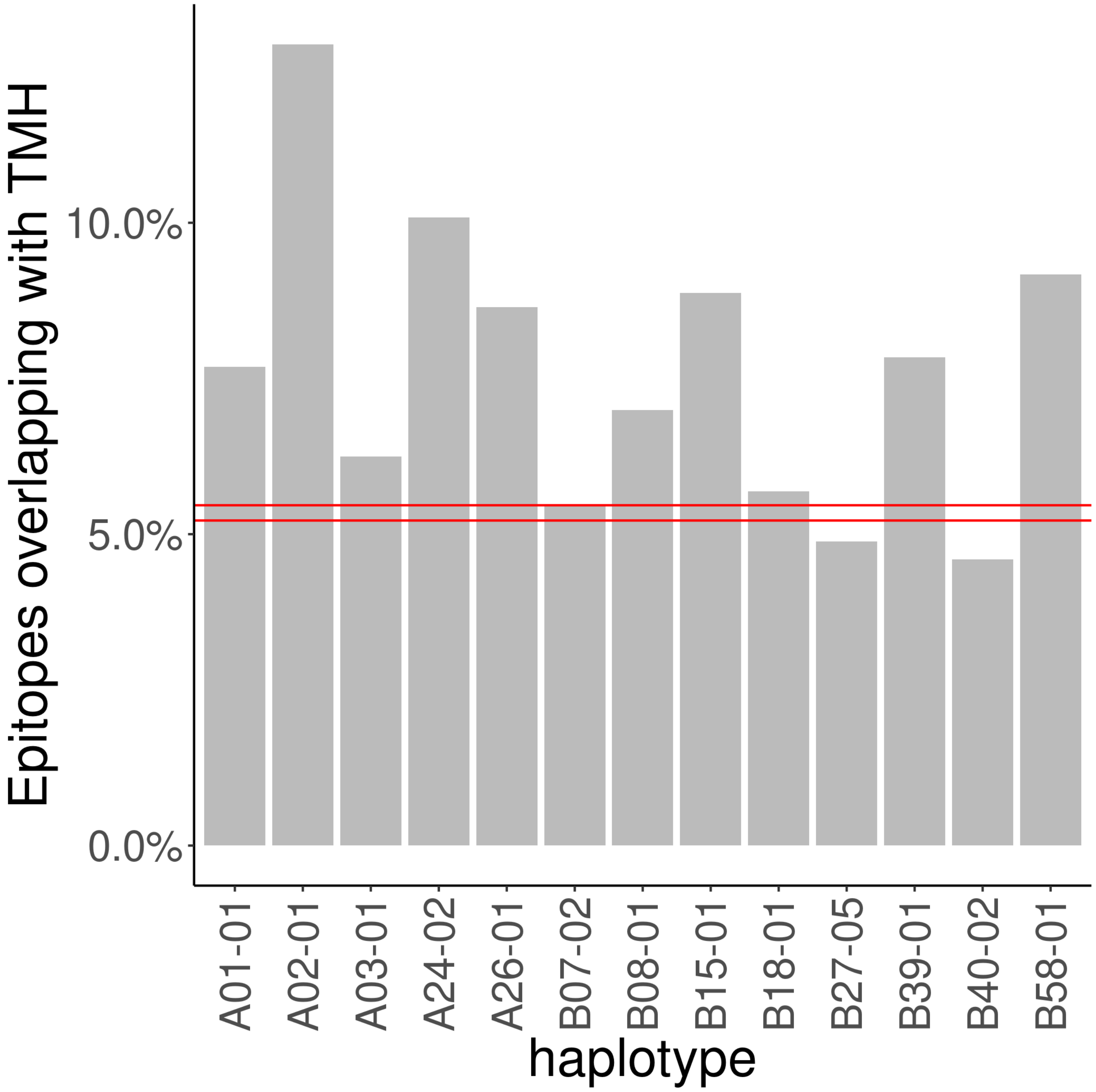

Figure S1. Results for Bianchi et al. (2017). Dashed lines denotes the coincidence interval.

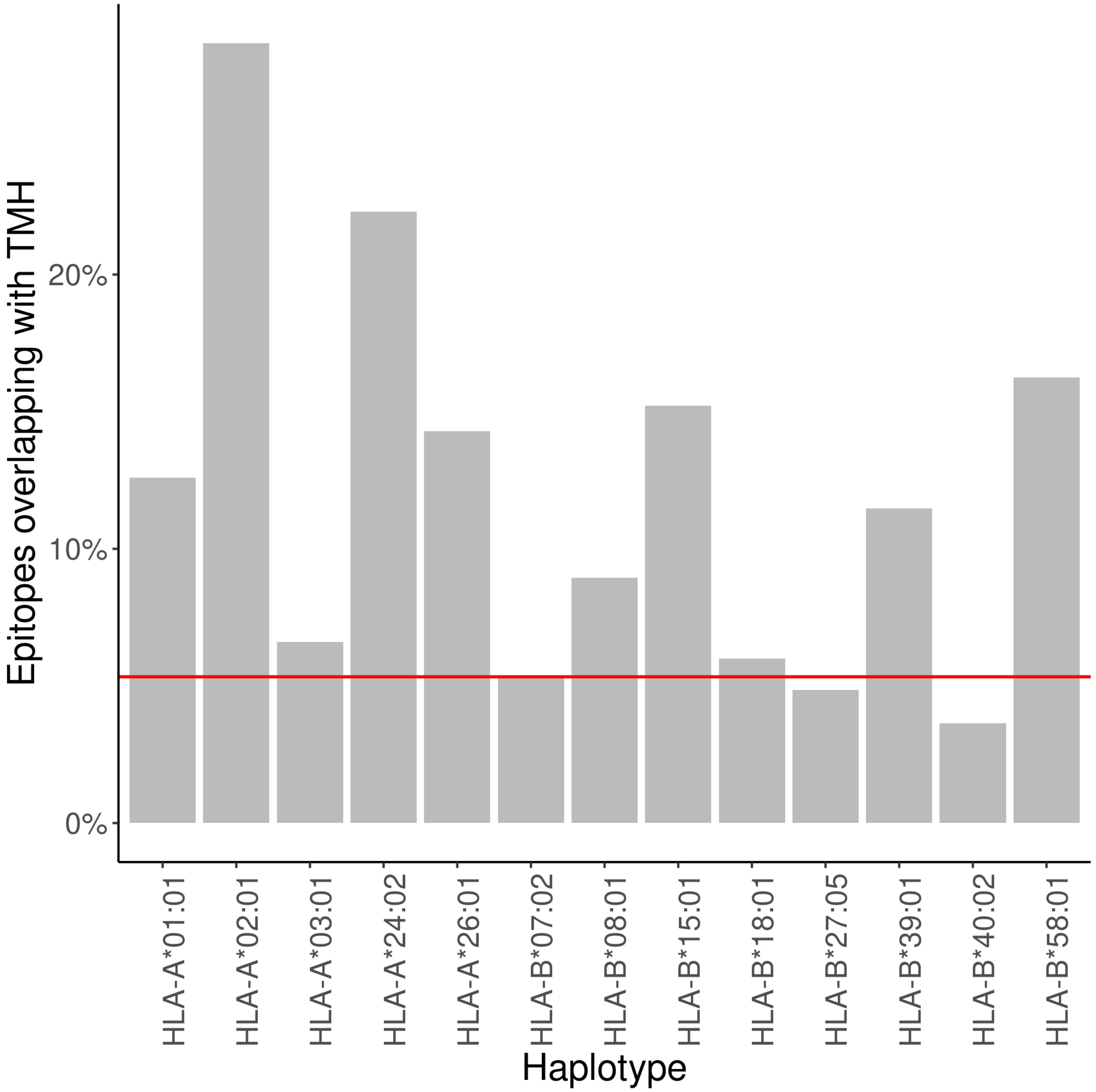

Figure S2. Results for this study. Dashed line denotes the percentage as expected by chance.

Table S1. IC50 values (in nM) per haplotype below which a peptide is considered a binder. percentage used: 2

haplotype	covid	human	myco
HLA-A*01:01	1470.5912	2545.9537	2812.1714
HLA-A*02:01	118.9596	218.7274	186.7565
HLA-A*03:01	537.0144	804.7455	1544.1073
HLA-A*24:02	984.8147	1590.0623	1971.8258
HLA-A*26:01	1095.2591	1771.6924	1526.1101
HLA-B*07:02	1215.7734	705.6514	435.5361
HLA-B*08:01	886.5661	883.0951	1023.2213
HLA-B*18:01	921.4157	1063.2215	1319.0445
HLA-B*27:05	1186.0963	689.8815	475.6130
HLA-B*39:01	437.3506	484.3843	399.3873
HLA-B*40:02	585.6308	541.2392	600.1688
HLA-B*58:01	435.4693	591.0526	538.9063
HLA-B*15:01	281.9129	440.6541	482.8369

Table S2. IC50 values (in nM) per haplotype below which a peptide is considered a binder. percentage used: 2

haplotype	covid	human	myco
HLA-DRB1*0101	7.3896	9.72	9.9600
HLA-DRB1*0301	121.8420	198.40	164.4900
HLA-DRB1*0401	59.8780	74.92	84.3112
HLA-DRB1*0405	46.2324	51.88	66.7100
HLA-DRB1*0701	17.7464	22.40	28.1700
HLA-DRB1*0802	99.7592	137.16	67.9900
HLA-DRB1*0901	42.3464	53.52	41.5400
HLA-DRB1*1101	35.9988	39.01	48.9200
HLA-DRB1*1201	194.4408	248.72	289.7300
HLA-DRB1*1302	21.1084	40.59	35.4100
HLA-DRB1*1501	32.6196	40.69	46.6700
HLA-DRB3*0101	175.2984	298.94	218.7300
HLA-DRB3*0202	176.8168	291.95	405.8724
HLA-DRB4*0101	47.6384	51.04	62.7800
HLA-DRB5*0101	32.8872	43.52	60.2312
HLA-DQA1*0501/DQB1*0201	193.1108	209.89	174.2124
HLA-DQA1*0501/DQB1*0301	51.2028	43.47	20.3200
HLA-DQA1*0301/DQB1*0302	361.8180	365.96	296.4712
HLA-DQA1*0401/DQB1*0402	214.1932	242.68	199.8912
HLA-DQA1*0101/DQB1*0501	550.4488	674.95	930.9612
HLA-DQA1*0102/DQB1*0602	157.4480	174.82	114.3512

2 IC50 VALUES OF BINDERS PER MHC ALLELE

Per target proteome (i.e. human, SARS-CoV-2, *M tuberculosis*), we collected all 9-mers (for MHC-I) and 14-mers (for MHC-II), after removing the selenoproteins and proteins that are shorter than the epitope length. From these epitopes, per MHC allele, we predicted the IC50 (in nM) using `epitope-prediction` (for MHC-I) and MHCnuggets (for MHC-II). Here, we show the IC50 value per MHC allele that is used to determine if a peptide binds to the allele's MHC for MHC-I (see supplementary Table S1) and MHC-II (see supplementary Table S2).

Table S3. Amounts. raw = all variations, including DNA variations. all_proteins = all proteins. map = membrane associated protein. tmp = transmembrane protein. in_tmh = in transmembrane helix of TMP. in_sol = in soluble region of TMP.

what	raw	all_proteins	map	tmp	in_tmh	in_sol
Number of variations	60931	37831	16623	21208	3803	17405
Number of unique variations	60544	37630	16606	21024	3789	17235
Number of unique SNPs	NA	9621	4219	6026	1140	4936
Number of unique gene names	953	911	457	605	325	590
Number of unique protein names	5163	4780	2227	2553	1280	2467
Percentage TMH	NA	10	0	19	26	18

Table S4. Amounts. single_in_tmh = in transmembrane helix of single-spanner. single_in_sol = in soluble region of single-spanner. multi_in_tmh = in transmembrane helix of multi-spanner. multi_in_sol = in soluble region of multi-spanner.

what	single_in_tmh	single_in_sol	multi_in_tmh	multi_in_sol
Number of variations	452	7734	3351	9671
Number of unique variations	451	7733	3338	9502
Number of unique SNPs	160	2393	994	2762
Number of unique gene names	96	282	243	344
Number of unique protein names	304	1032	976	1435
Percentage TMH	11	5	35	26

3 COUNTS

See supplementary Tables S3 and S4 for an overview of all amounts. Note that, for the analyses using the SARS-CoV-2 virus proteome, we labeled this by its disease (`covid`) to prevent typos. In supplementary Table S3 there are multiple instances where the amounts are expected to add up, yet don't, as one SNP can work on multiple isoforms. For example, there are 9,621 unique SNPs found in all proteins, of which 4,219 around found in MAPs and 6,026 in TMPs. Apparently, 624 SNPs work on a set of isoforms that contains both MAPs and TMPs.

4 RELATIVE POSITIONS

See Supplementary Figure S3 for the distribution of the relative position of the SNPs.

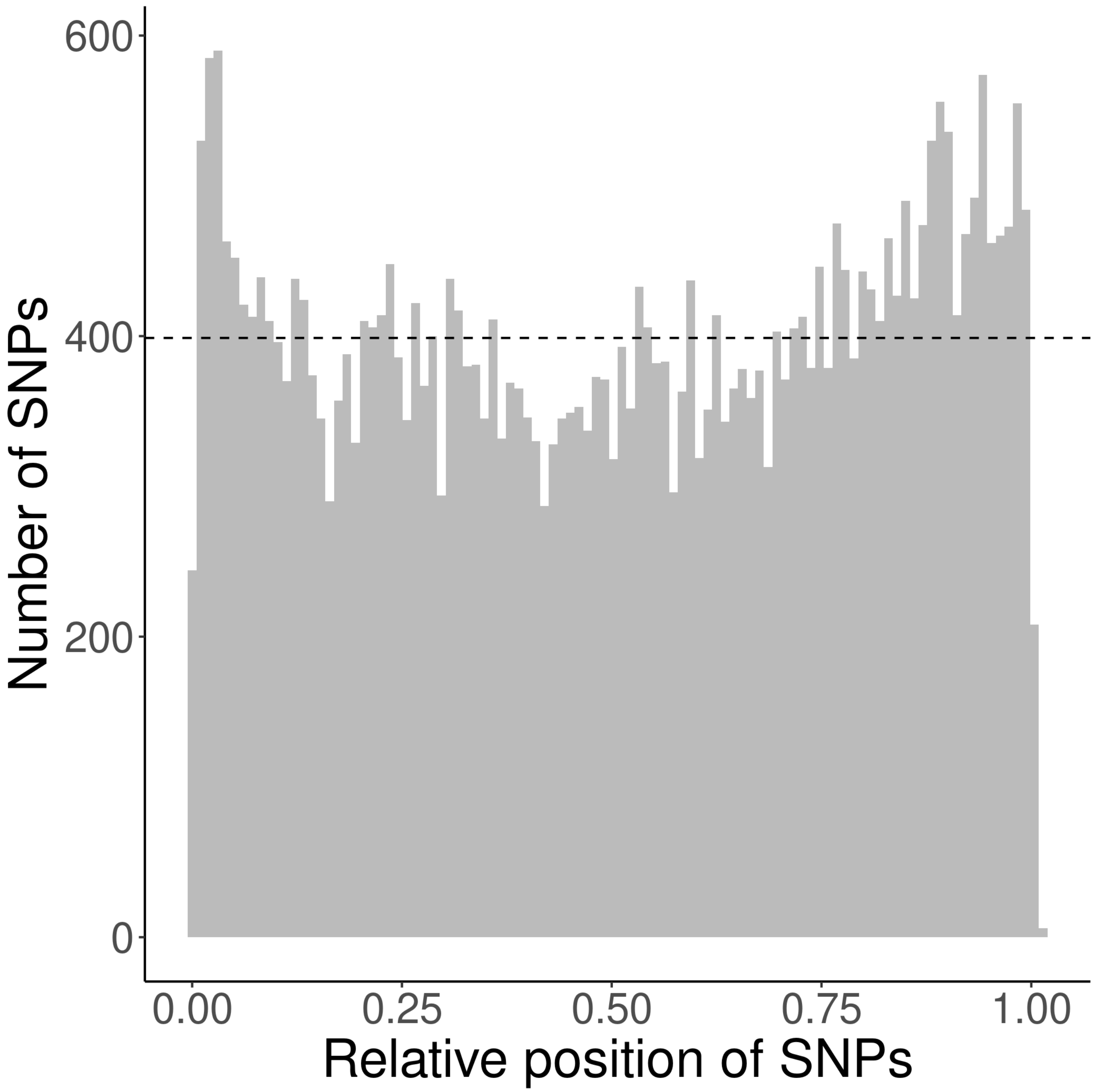

Figure S3. Distribution of the relative position of the SNPs used, where a relative position of zero denotes the first amino acid at the N-terminus, where a relative position of one indicates the last residue at the C-terminus.

Table S5. Percentage of MHC-II 14-mers overlapping with TMH. Values in brackets show the number of binders that have at least one residue overlapping with a TMH (first value)as well as the number of binders (second value). percentage used: 2

haplotype	covid	human	myco
HLA-DQA1*0101/DQB1*0501	40.433 (112/277)	31.214 (69752/223464)	32.158 (8187/25459)
HLA-DQA1*0102/DQB1*0602	22.910 (74/323)	16.167 (35753/221147)	17.950 (4608/25671)
HLA-DQA1*0301/DQB1*0302	10.381 (30/289)	10.179 (22623/222248)	11.144 (2842/25502)
HLA-DQA1*0401/DQB1*0402	11.111 (32/288)	13.135 (29319/223219)	9.890 (2524/25522)
HLA-DQA1*0501/DQB1*0201	20.430 (57/279)	16.240 (36186/222820)	14.999 (3823/25489)
HLA-DQA1*0501/DQB1*0301	15.808 (46/291)	14.106 (31046/220089)	18.969 (4878/25715)
HLA-DRB1*0101	27.119 (80/295)	19.774 (43968/222349)	22.293 (5692/25533)
HLA-DRB1*0301	14.676 (43/293)	9.801 (21831/222752)	7.956 (2025/25451)
HLA-DRB1*0401	19.231 (55/286)	15.325 (34011/221930)	18.113 (4641/25623)
HLA-DRB1*0405	12.996 (36/277)	13.684 (30380/222012)	15.837 (4036/25484)
HLA-DRB1*0701	32.877 (96/292)	21.512 (47856/222465)	29.304 (7471/25495)
HLA-DRB1*0802	23.132 (65/281)	19.339 (42859/221623)	28.805 (7358/25544)
HLA-DRB1*0901	11.565 (34/294)	13.111 (29043/221520)	16.798 (4301/25605)
HLA-DRB1*1101	25.197 (64/254)	11.924 (26582/222928)	16.103 (4101/25467)
HLA-DRB1*1201	36.897 (107/290)	15.482 (34596/223464)	20.018 (5098/25467)
HLA-DRB1*1302	13.962 (37/265)	20.121 (44798/222646)	23.141 (5935/25647)
HLA-DRB1*1501	35.206 (94/267)	21.836 (48671/222893)	25.891 (6584/25430)
HLA-DRB3*0101	9.158 (25/273)	8.496 (18884/222274)	6.819 (1740/25517)
HLA-DRB3*0202	18.657 (50/268)	13.832 (30687/221859)	15.843 (4059/25620)
HLA-DRB4*0101	23.529 (68/289)	12.749 (28376/222568)	16.221 (4131/25467)
HLA-DRB5*0101	23.776 (68/286)	11.235 (24993/222464)	14.648 (3732/25478)

5 PRESENTATION OF TMH-DERIVED EPITOPES

See supplementary Table S5 for the percentage of MHC-II 14-mers overlapping with TMH.

6 THE PERCENTAGE OF TMH-DERIVED EPITOPES FROM IEDB EPITOPES

We display the over-presentation of epitopes taken from the IEDB database, for two assays: an MHC ligand assay (Figure 2A) and a T cell assay (see figure S5), as a bar plot. Supplementary Table S6 below shows the exact numbers.

MHC class	Dataset	n
I	iedb_mhc_ligand	22.28% (1789/8030)
I	iedb_t_cell	35.91% (93/259)
II	iedb_mhc_ligand	10.46% (73/698)
II	iedb_t_cell	6.66% (42/631)

Table S6. Percentage of epitopes derived from a TMH for epitopes taken from the IEDB, for two different types of assays: an MHC ligand assay, as well as a T cell assay. The values between brackets show the the number of epitopes that were predicted to overlapping with a TMH per all epitopes that could be uniquely mapped to the representative human reference proteome.

7 CORRELATION OF EPITOPE PRESENTATION

In the main text of this research, we use two sources of epitopes to determine if TMH-derived epitopes are presented. The first source of epitopes are all the 9-mers (for MHC-I) (and 14-mers for MHC-II) derived from a human reference proteome, where this over-presentation is displayed in figure 1A. The second source of epitopes are those that are present in the IEDB that are obtained from MHC ligand assays, as displayed in figure 2A.

Here we correlate between the over-presentation of TMH-derived epitopes between these two sources of data. Figure S4 shows per MHC allele the percentage of TMH-derived epitopes, with a linear trendline.

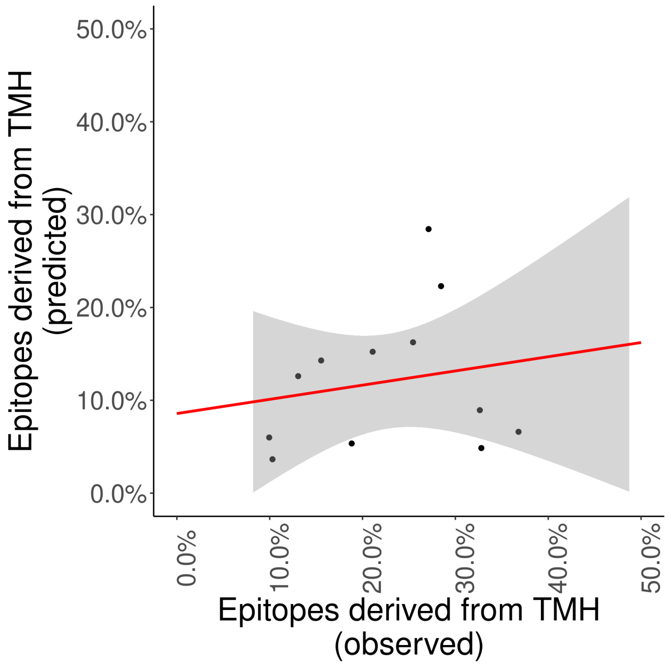

Figure S4. TMH-derived epitopes are over-presented when using predicted as well as experimental data For the MHC class I alleles, the over-presentation of TMH-derived epitopes is correlated between IEDB MHC ligand epitopes (horizontal axis) and the 9-mers derived from a human reference proteome (vertical axis). Alleles are listed in Table S8). The trendline shows the linear correlation between these percentages, where the gray area is the 95% confidence interval.

8 PRESENTATION OF TMH-DERIVED EPITOPES RESULT IN T CELL RESPONSES

Figure S5 shows the percentage of TMH-derived epitopes of the reported epitopes from human origin for which T-cell responses were established. The data was obtained from the IEDB and includes only the MHC alleles used in this study. As there are many (especially class II) MHC alleles, only a small percentage of the full IEDB data could be used.

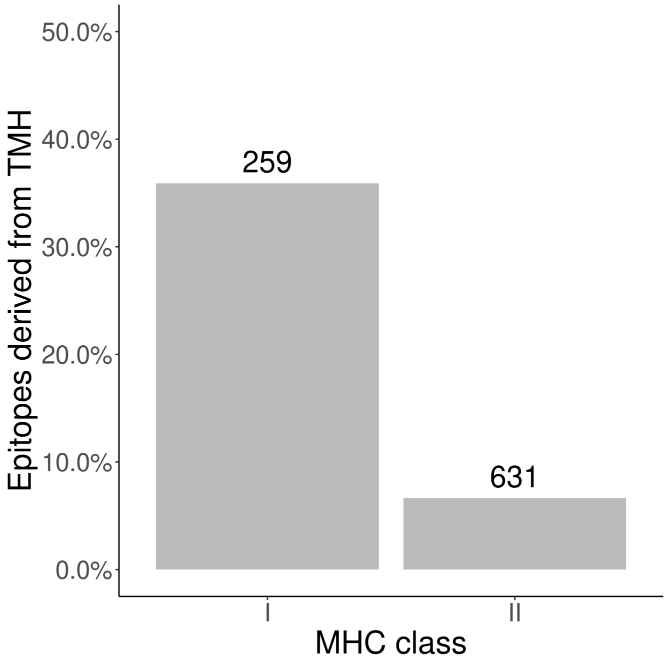

Figure S5. TMH-derived epitopes evoke T-cell responses The numbers above the bars denotes the number of epitopes found in the IEDB for the MHC alleles used in this study.

Table S7. Percentage of MHC-I 9-mers overlapping with TMH. Values in brackets show the number of binders that have at least one residue overlapping with a TMH (first value)as well as the number of binders (second value). percentage used: 2

haplotype	covid	human	myco
HLA-A*01:01	15.603 (44/282)	12.600 (28377/225209)	11.424 (2947/25797)
HLA-A*02:01	34.155 (97/284)	28.441 (63994/225003)	29.749 (7646/25702)
HLA-A*03:01	9.122 (27/296)	6.606 (14851/224796)	9.972 (2565/25721)
HLA-A*24:02	39.223 (111/283)	22.297 (50313/225648)	22.346 (5752/25741)
HLA-A*26:01	21.739 (65/299)	14.287 (32232/225598)	13.950 (3598/25793)
HLA-B*07:02	9.712 (27/278)	5.347 (11893/222429)	8.899 (2291/25744)
HLA-B*08:01	15.248 (43/282)	8.935 (19981/223616)	10.714 (2750/25667)
HLA-B*15:01	24.324 (72/296)	15.228 (34498/226542)	17.600 (4547/25835)
HLA-B*18:01	11.724 (34/290)	5.993 (13409/223745)	5.960 (1536/25773)
HLA-B*27:05	10.227 (27/264)	4.854 (10882/224178)	8.031 (2063/25688)
HLA-B*39:01	18.182 (50/275)	11.468 (25621/223419)	14.682 (3787/25793)
HLA-B*40:02	4.594 (13/283)	3.647 (8147/223408)	4.264 (1097/25729)
HLA-B*58:01	24.731 (69/279)	16.245 (36409/224119)	20.558 (5292/25742)

9 PRESENTATION OF TMH-DERIVED EPITOPES

See supplementary Table S7 for the percentage of MHC-I 9-mers overlapping with TMH.

Supplementary Table S8 shows the shorthand notation for the HLA alleles.

Supplementary Tables S7 and S5 show the exact number of binders, binders that overlap with TMHs and the percentage of binders that overlap with TMHs, as visualized by figure 1A.

index	haplotype_name
1	HLA-A*01:01
2	HLA-A*02:01
3	HLA-A*03:01
4	HLA-A*24:02
5	HLA-A*26:01
6	HLA-B*07:02
7	HLA-B*08:01
8	HLA-B*18:01
9	HLA-B*27:05
10	HLA-B*39:01
11	HLA-B*40:02
12	HLA-B*58:01
13	HLA-B*15:01
1	HLA-DRB1*0101
2	HLA-DRB1*0301
3	HLA-DRB1*0401
4	HLA-DRB1*0405
5	HLA-DRB1*0701
6	HLA-DRB1*0802
7	HLA-DRB1*0901
8	HLA-DRB1*1101
9	HLA-DRB1*1201
10	HLA-DRB1*1302
11	HLA-DRB1*1501
12	HLA-DRB3*0101
13	HLA-DRB3*0202
14	HLA-DRB4*0101
15	HLA-DRB5*0101
16	HLA-DQA1*0501/DQB1*0201
17	HLA-DQA1*0501/DQB1*0301
18	HLA-DQA1*0301/DQB1*0302
19	HLA-DQA1*0401/DQB1*0402
20	HLA-DQA1*0101/DQB1*0501
21	HLA-DQA1*0102/DQB1*0602

Table S8. Abbreviations of the haplotype names

Goal	Tool	Reference
Predict topology	TMHMM	Krogh et al. (2001)
Predict topology	PureseqTM	Wang et al. (2019)
Predict epitopes MHC-I	`epitope-prediction`	Bianchi et al. (2017)
Predict epitopes MHC-II	NetMHCIIpan	Nielsen et al. (2008); Karosiene et al. (2013)
Call TMHMM from R	`tmhmm`	Bilderbeek (2019b)
Call PureseqTM from R	`pureseqtmr`	Bilderbeek (2020b)
Call NetMHCIIpan from R	`netmhc2pan`	Bilderbeek (2019a)
Work with IEDB	`iedbr`	Bilderbeek (2021a)
Work with `rentrez`	`sprentrez`	Bilderbeek (2021b)
Combine all	`bbbq`	Bilderbeek (2020a)

Table S9. Overview of all software used in this research.

10 PREDICTION SOFTWARE USED

For this research, we needed software to predict protein topology, as well as the MHC-I and MHC-II binding affinities of epitopes. We selected our software, by searching the scientific literature to identify the most recent free and open source (FOSS) prediction software. This was done by searching for papers that (1) cite older prediction software, and (2) present a novel method to make predictions. As a starting point, per type of prediction software, a review paper was used (Möller et al. (2001) for protein topology, Lundegaard et al. (2011) for MHC-I binding affinities and Nielsen et al. (2003) for MHC-II binding affinities).

There are multiple computational tools developed to predict which parts of a protein forms a TMH. In 2001, multiple of such prediction tools have been compared Möller et al. (2001), of which TMHMM Krogh et al. (2001) turned out to be the most accurate, as is used in the previous study Bianchi et al. (2017). However, TMHMM has a restrictive software license and is nearly two decades old. Therefore, PureseqTM Wang et al. (2019), was also used in this study, which has been more recently developed and has a free software license.

For MHC-I, there are multiple computational tools developed to predict epitopes. According to Lundegaard et al. (2011), at that time, NetMHCcons Karosiene et al. (2012) gave the best predictions. We used the same tool as used in our earlier study, `epitope-prediction` Bianchi et al. (2017),

Also for MHC-II, there are multiple computational tools developed to predict epitopes, such as using a trained neural network Nielsen et al. (2003) or a Gibbs sampling approach Nielsen et al. (2004). According to Lundegaard et al. (2011), in 2011, from a set of multiple tools, NetMHCIIpan Nielsen et al. (2008); Karosiene et al. (2013) made the most accurate predictions. The most recent FOSS tool available now appears to be MHCnuggets Shao et al. (2020), which can do both MHC-I and MHC-II predictions. As we already use `epitope-prediction` Bianchi et al. (2017) for MHC-I predictions, we use MHCnuggets only for MHC-II predictions.

To retrieve the data from the NCBI databases the `rentrez` R package Winter (2017) was used that calls the NCBI database's API. The NCBI database provides a stable user experience for all users, by limiting its API to 3 calls per second per user. Additionally, the API splits the result of a bigger query into multiple pages, each of which needs one API call. The `sprentrez` package Bilderbeek (2021b) provides for bigger queries of multiple (and delayed) API calls.

To retrieve the data from the IEDB databases Vita et al. (2019), the `iedbr` R package Bilderbeek (2021a) was written, to calls the IEDB database's API. Similar to the NCBI database, the IEDB has a limit to 1

call per second per user and allows a query results to return 10k results maximally. The `iedbr` package Bilderbeek (2021a) allows for bigger queries.

11 PREDICTION SOFTWARE WRITTEN

The R programming language is used for the complete experiment, including the analysis. The complete experiment is bundled in the 'bbbq' R package, which is dependent on 'tmhmm', 'pureseqtmr', 'epitope-prediction' and 'mhcnuggetsr' as described below.

The R package 'tmhmm' was developed to do the similar topology predictions as our earlier study (that used 'TMHMM'), yet in an automated way. 'TMHMM' has a restrictive software license Krogh et al. (2001) and allows a user to download a pre-compiled executable after confirmation that he/she is in academia. The R package respects this restriction and allows the user to install and use TMHMM from within R, as done in this study. 'tmhmm' has been submitted to and is accepted by the Comprehensive R Archive Network (CRAN).

To be able to call, from R, the TMH prediction software 'PureseqTM' Wang et al. (2019), which is written in C, the package 'pureseqtmr' has been developed. 'pureseqtmr' allows to install 'PureseqTM' and use most of its features. 'pureseqtmr' has been submitted to and is accepted by CRAN.

MHCnuggets is a free and open-source Python package to predict epitope affinity for many MHC-I and MHC-II variants Shao et al. (2020). The R package 'mhcnuggetsr' allows one to install and use MHCnuggets from within R. Also 'mhcnuggetsr' has been submitted to and is accepted by CRAN.

To reproduce the full experiment presented in this paper, the functions needed are bundled in the 'bbbq' R package. This package is too specific to be submitted to CRAN.

Table S10. Percentage of spots and spots that overlap with a TMH

target	mhc_class	n_spots	n_spots_tmh	f_tmh
covid	1	14207	1124	7.91
covid	2	14137	1245	8.81
human	1	11220940	598391	5.33
human	2	11118448	672273	6.05
myco	1	1299707	98613	7.59
myco	2	1279742	108419	8.47

12 PREDICTION OF PERCENTAGE OF EPITOPES OVERLAPPING WITH A TMH

Supplementary Table S10 shows an overview of the findings, where a target specifies the source of the proteome, where `covid` denotes SARS-CoV-2 and `myco` denotes *Mycobacterium tuberculosis*. `mhc_class` denotes the MHC class, `n_spots` the number of possible 9-mers (for MHC-I) or 14-mers (for MHC-II) possible. `n_spots_tmh` the number of epitopes that overlapped with a TMH that were binders. `f_tmh` the percentage of peptides that had at least 1 residue overlapping with a TMH.

13 MINOR METHODS

These are details that are removed from the 'Methods' section.

PureseqTM does not predict the topology of proteins that have less than three amino acids. The TRDD1 ('T cell receptor delta diversity 1') protein, however, is two amino acids long. The R package `pureseqtmr`, however, predicts that mono- and di-peptides are cytosolic.

14 MINOR DISCUSSION

These are details that are removed from the 'Discussion' section.

In this experiment we predicted epitopes that overlap with TMHs from a human, bacterial and viral proteome, would these proteins be expressed in a human host. Bacteria, however have different cell membranes and cell walls, hence different structural requirements for a TMH. Both topology prediction tools were trained to recognize human TMHs, thus we cannot be sure that the transmembrane regions predicted in bacterial proteins are actually part of a TMH. For the purpose of this study, we assume the error in topology predictions to be unbiased way towards topology. In other words: that a bacterial TMH is incorrectly predicted to be absent just as often as it is incorrectly predicted to be present elsewhere.

Regarding the evolutionary conservation of TMHs using SNPs, again, it is estimated that approximately ten percent of SNPs is a false positive that result from the methods to determine a SNP. One example is that sequence variations are incorrectly detected due to highly similar duplicated sequences Musumeci et al. (2010). We assume that these duplications occur as often in TMHs as in regions around these, hence we expect this not to affect our results.

In our evolutionary experiment, we removed variations that were synonymous mutations (i.e. resulted in the same amino acid, from a different genetic code) from our analysis. There is evidence, however, that these synonymous mutations do have an effect and may even be evolutionary selected for Hunt et al. (2009). As the possible effect of synonymous mutations is ignored by our topology prediction software, we do so as well.

15 RELATIVE PRESENTATION OF TMH-DERIVED EPITOPES

To compare the over-presentation of TMH-derived epitopes between the different proteomes, we normalized this percentages in such a way that 1.0 is the percentage of TMH-derived epitopes that would be expected by chance. Figure S6 and S7 show these normalized values for the MHC-I and MHC-II alleles respectively.

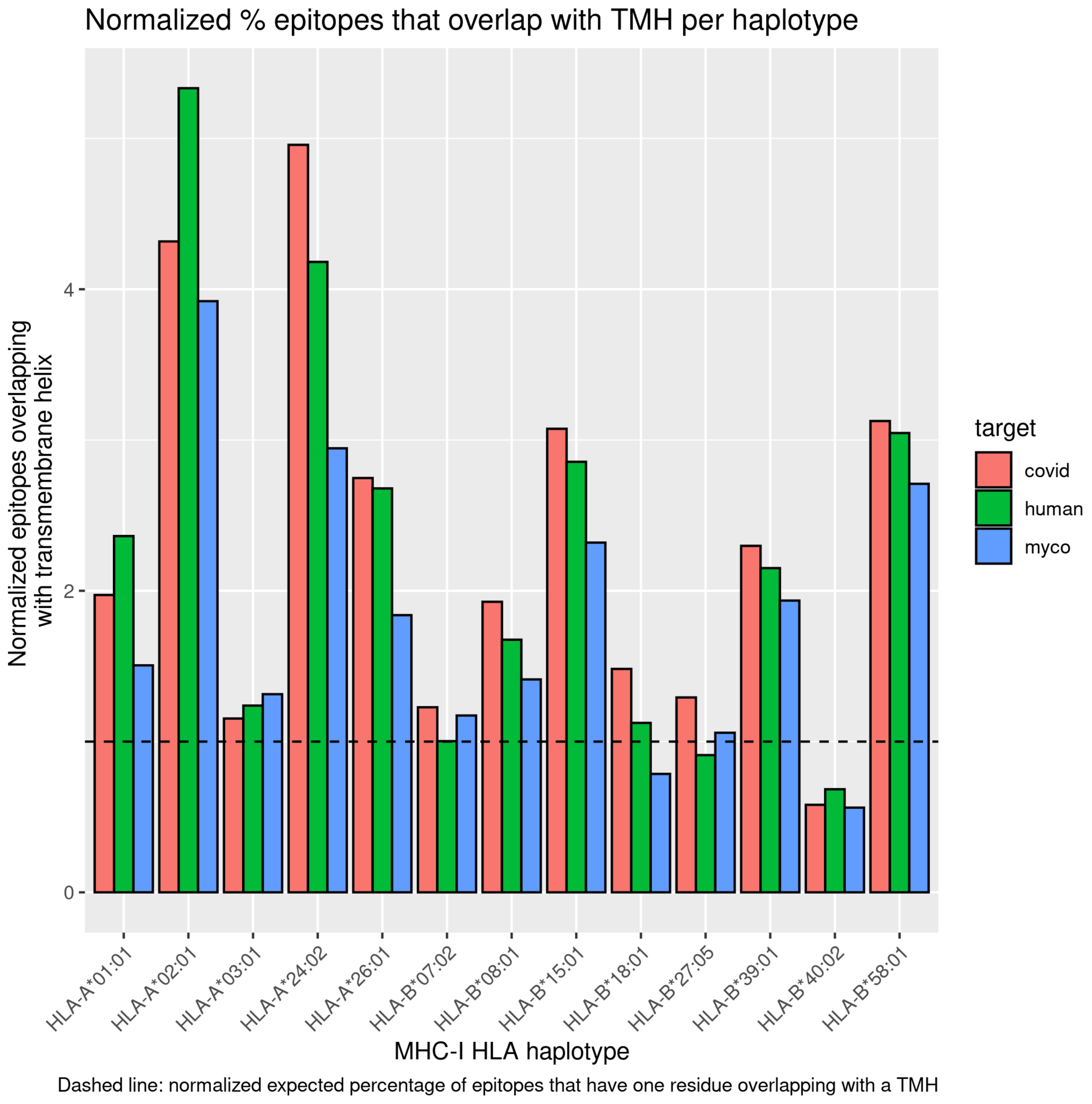

Figure S6. Normalized proportion of MHC-I epitopes overlapping with TMHs for human, viral and bacterial proteomes. Legend: covid = SARS-CoV-2, human = *Homo sapiens*, myco = *Mycobacterium tuberculosis*

To determine the additional over-presentation of TMH-derived epitopes in MHC-II (as compared to MHC-I), we normalized the data to enable a side-by-side comparison. The percentage of TMH-derived

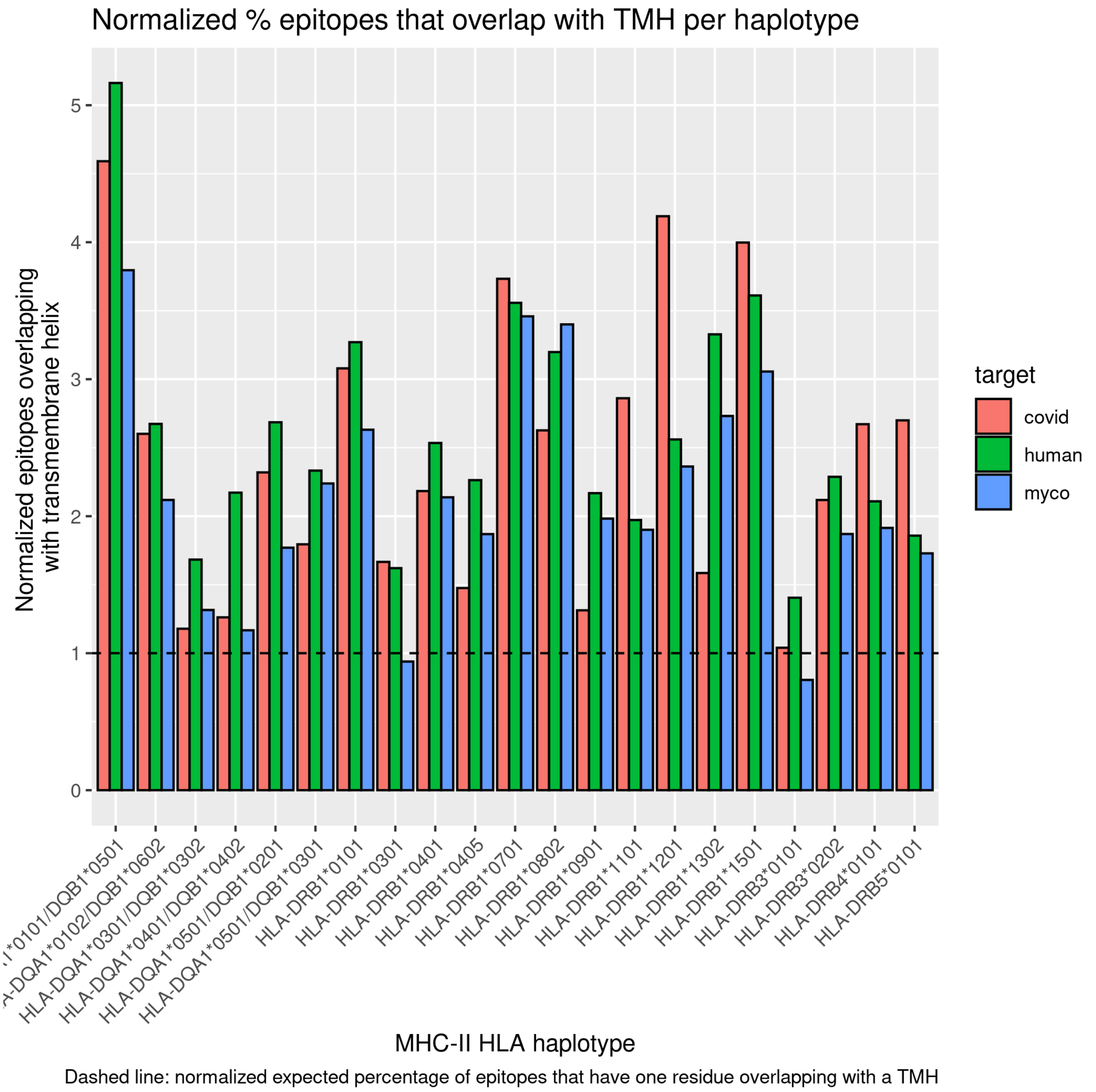

Figure S7. Normalized proportion of MHC-II epitopes overlapping with TMHs for human, viral and bacterial proteomes. Legend: covid = SARS-CoV-2, human = *Homo sapiens*, myco = *Mycobacterium tuberculosis*

epitopes presented was normalized to the expected percentage of TMH-derived epitopes, where 1.0 denotes that the percentage of presented TMH-derived epitopes matches the values as expected by chance. The normalized values per MHC allele are shown in figure S8. To compare the TMH-derived over-presentation per MHC class, we grouped the normalized values per allele, and plot the mean and standard error, as shown in figure S9.

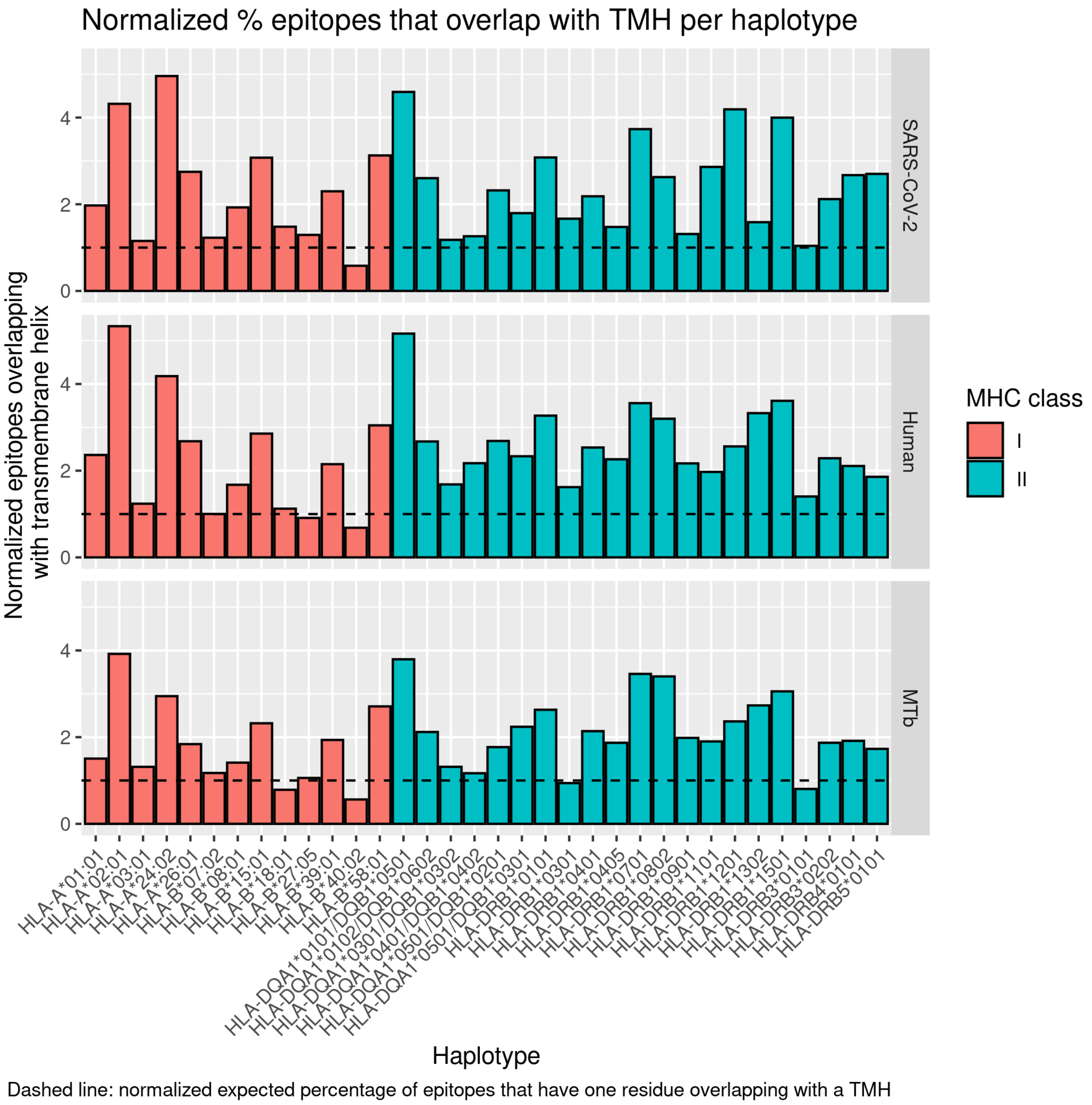

Figure S8. Normalized proportion of MHC-I and MHC-II epitopes overlapping with TMHs, for the different MHC alleles and proteomes

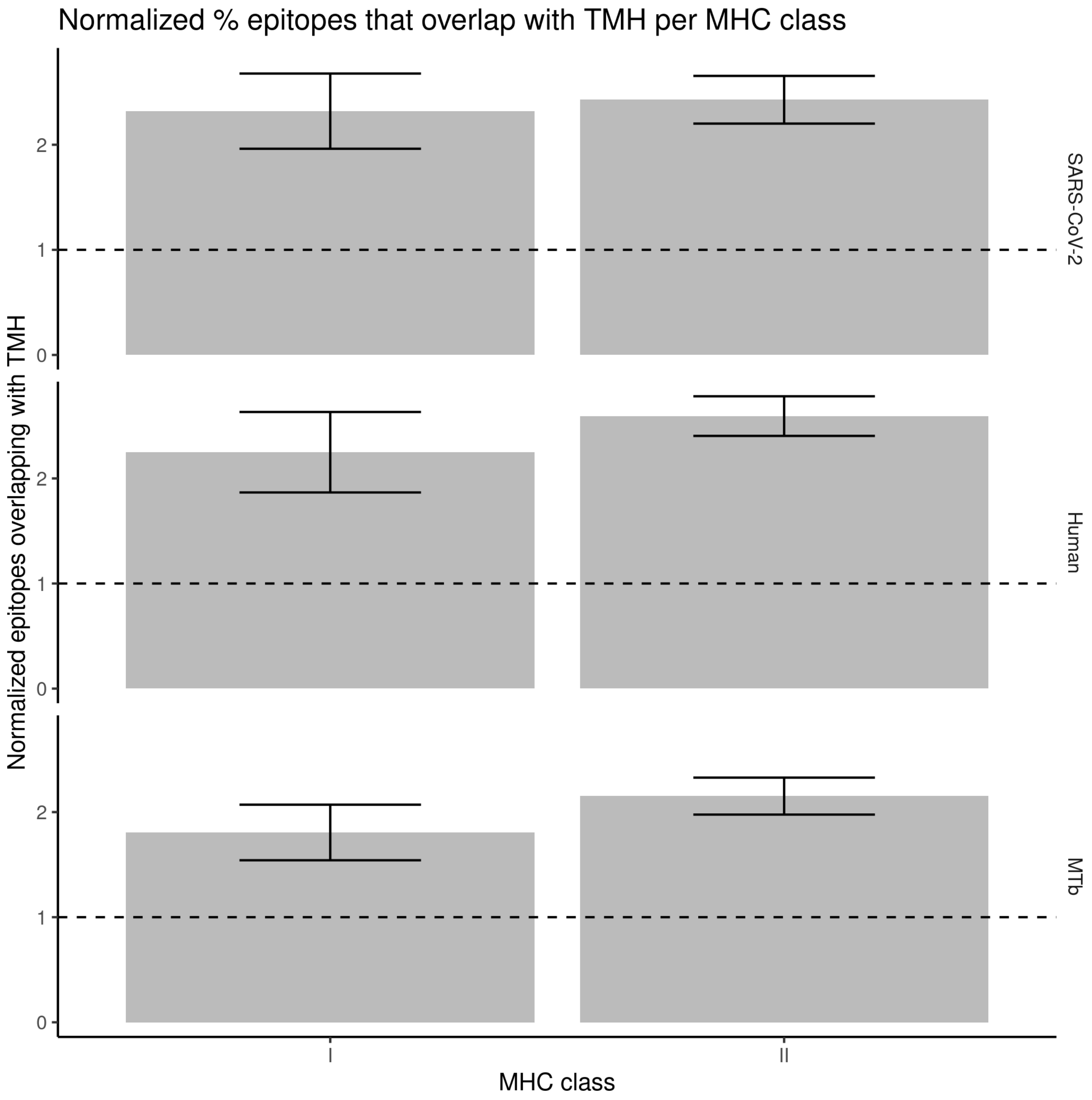

Figure S9. Normalized proportion of MHC-I and MHC-II epitopes overlapping with TMHs, for the different MHC classes and proteomes. Error bars denote the standard error.

16 EVOLUTIONARY CONSERVATION

Figure S10 shows the distribution of the number of SNPs per gene name, at the date we started the experiment, at December 14th 2020.

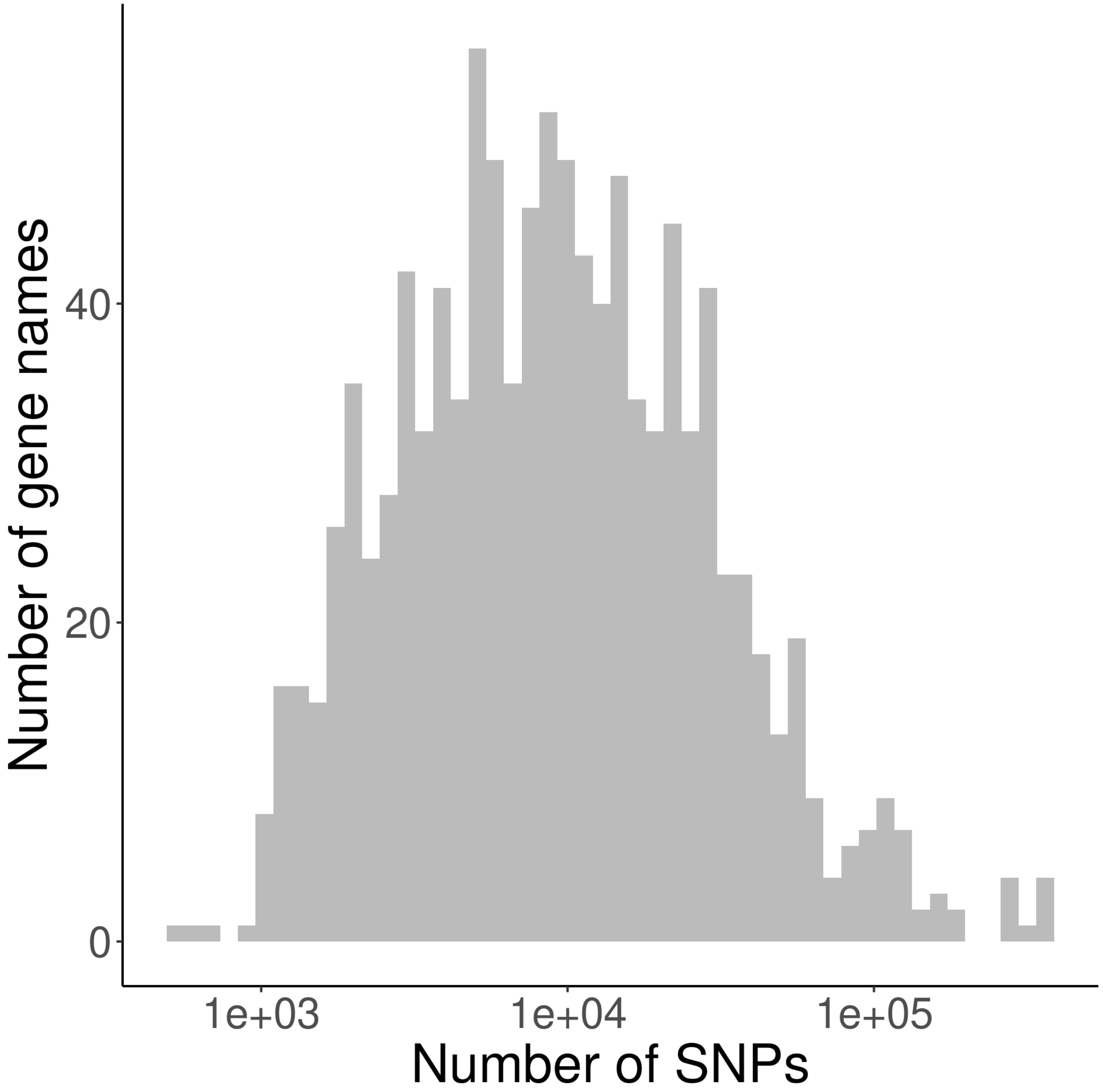

Figure S10. Distribution of the number of SNPs per gene name in the NCBI database.

To verify if SNPs were sampled uniformly over proteins, we show the distribution of the relative position in figure S3. We find no clear evidence of a bias.

Supplementary Table S11 shows the statistics for all SNPs, where supplementary Tables S12 and S13 show the statistics for only single-spanners and multi-spanners respectively.

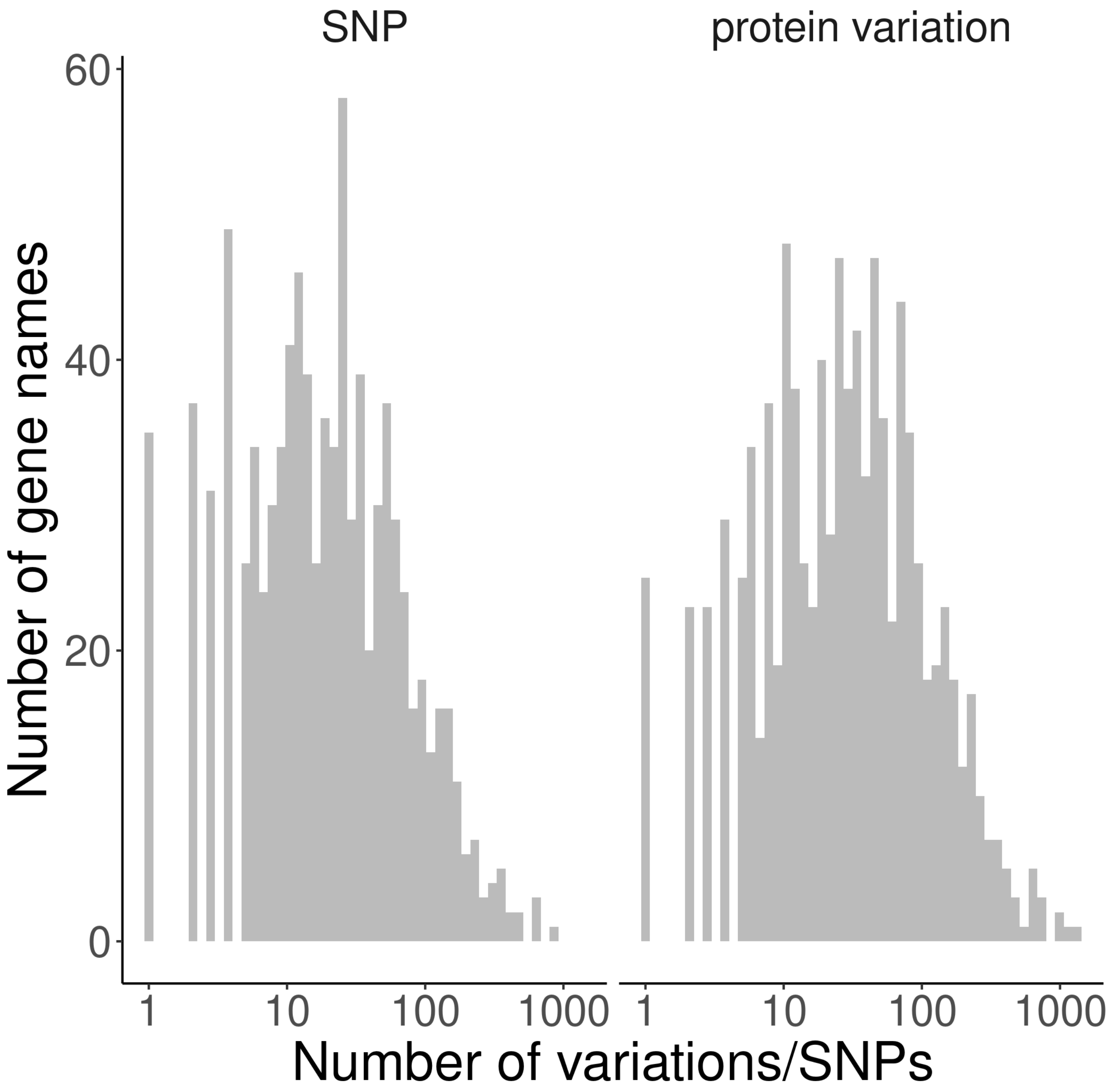

Figure S11. Distribution of the number of protein variations and SNPs per gene name processed.

Table S11. Statistics for all TMPs. p = p value. n = number of SNPs. n_success = number of SNPs found in TMHs (dashed blue line). E(n_success) = expected number of SNPs to be found in TMHs (dashed line).

parameter	value
p	6.820823e-11
n	21208
n_success	3803
E(n_success)	4140.56

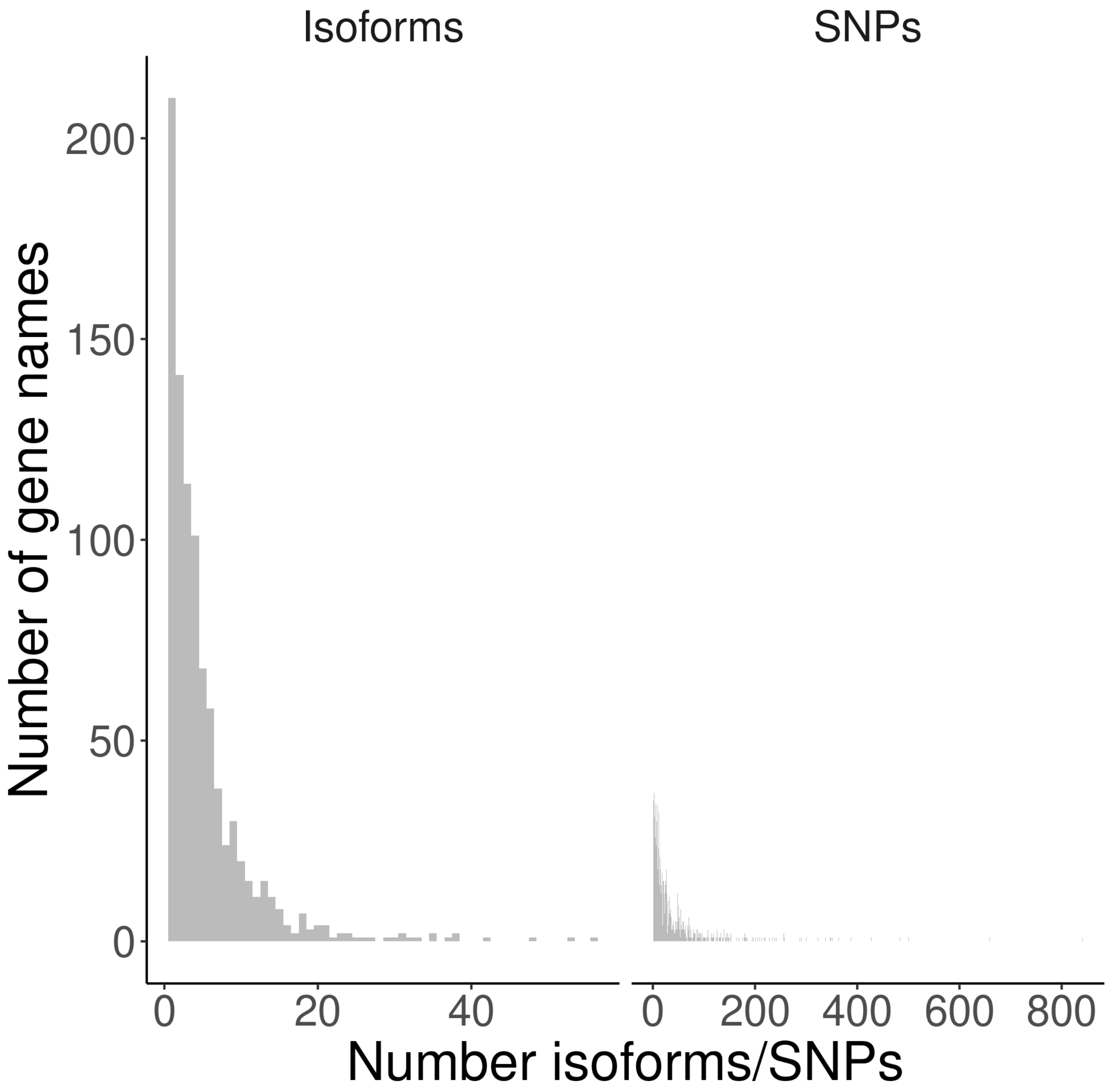

Figure S12. Histogram of the number of proteins found per gene name. Most often, a gene name is associated with one proteins.

Table S12. Statistics for the single-spanners. p = p value. n = number of SNPs in single-spanners. n_success = number of SNPs found in TMHs of single-spanners (dashed blue line). E(n_success) = expected number of SNPs to be found in TMHs of single-spanners (dashed line).

parameter	value
p	0.3189532
n	8186
n_success	452
E(n_success)	462.1535

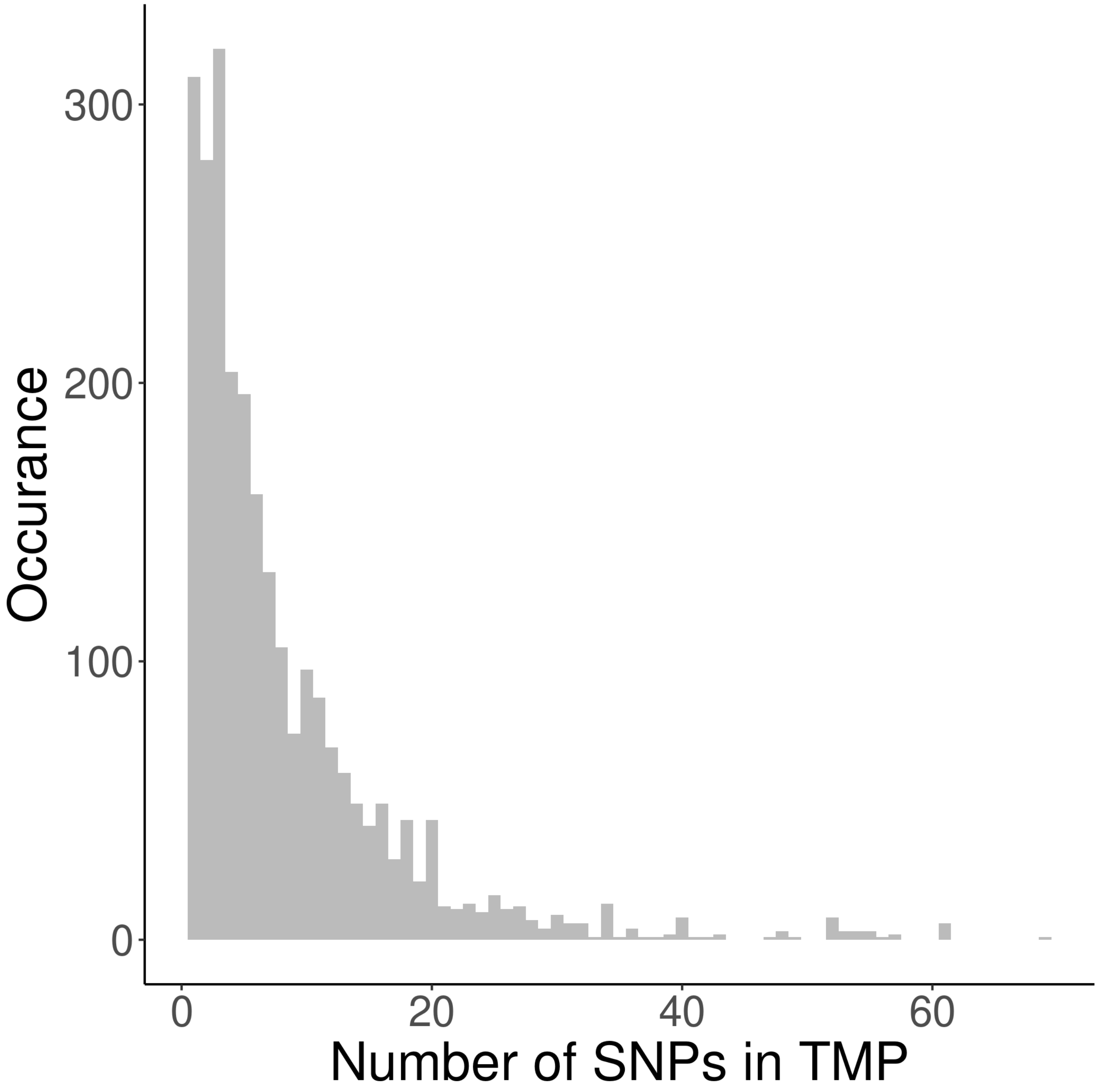

Figure S13. Histogram of the number of SNPs per trans-membrane protein. Dashed vertical line: average number of SNPs per TMP

Table S13. Statistics for the multi-spanners. p = p value. n = number of SNPs in multi-spanners. n_success = number of SNPs found in TMHs of multi-spanners (dashed blue line). E(n_success) = expected number of SNPs to be found in TMHs of multi-spanners (dashed line).

parameter	value
p	8.315841e-12
n	13022
n_success	3351
E(n_success)	3678.406

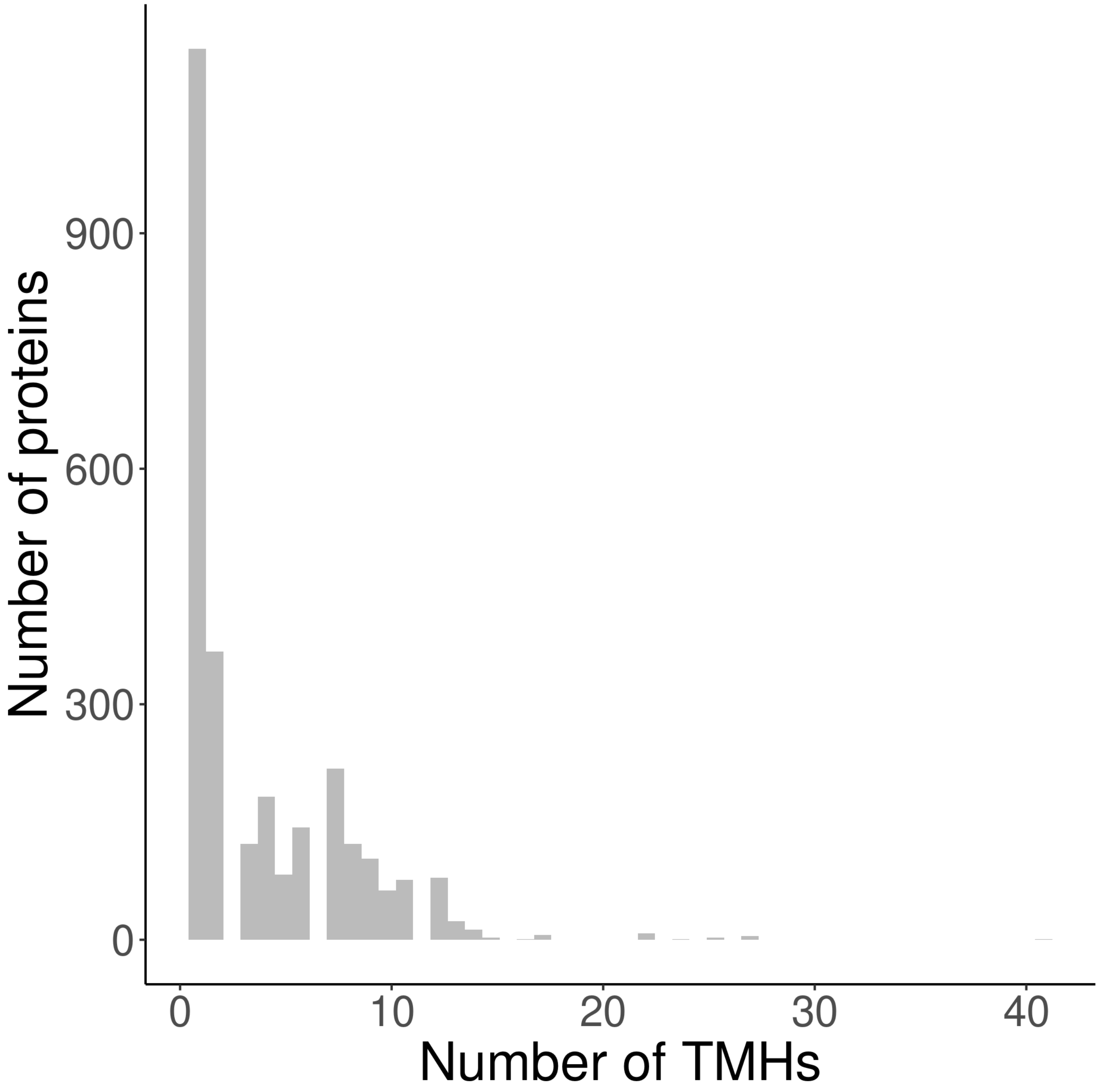

Figure S14. Histogram of the number of TMHs predicted per protein, for the trans-membrane proteins used.

Table S14. Percentage of MHC-II 14-mers overlapping with TMH. Values in brackets show the number of binders that have at least two residues overlapping with a TMH (first value)as well as the number of binders (second value). percentage used: 2

haplotype	covid	human	myco
HLA-DQA1*0101/DQB1*0501	39.711 (110/277)	30.813 (68855/223464)	31.777 (8090/25459)
HLA-DQA1*0102/DQB1*0602	22.910 (74/323)	15.858 (35070/221147)	17.713 (4547/25671)
HLA-DQA1*0301/DQB1*0302	10.381 (30/289)	9.996 (22217/222248)	10.960 (2795/25502)
HLA-DQA1*0401/DQB1*0402	11.111 (32/288)	12.915 (28829/223219)	9.670 (2468/25522)
HLA-DQA1*0501/DQB1*0201	20.072 (56/279)	15.969 (35582/222820)	14.830 (3780/25489)
HLA-DQA1*0501/DQB1*0301	15.808 (46/291)	13.890 (30570/220089)	18.682 (4804/25715)
HLA-DRB1*0101	27.119 (80/295)	19.401 (43139/222349)	21.944 (5603/25533)
HLA-DRB1*0301	13.993 (41/293)	9.415 (20972/222752)	7.638 (1944/25451)
HLA-DRB1*0401	19.231 (55/286)	14.925 (33122/221930)	17.652 (4523/25623)
HLA-DRB1*0405	12.635 (35/277)	13.298 (29523/222012)	15.469 (3942/25484)
HLA-DRB1*0701	32.192 (94/292)	21.057 (46845/222465)	28.884 (7364/25495)
HLA-DRB1*0802	23.132 (65/281)	18.909 (41907/221623)	28.496 (7279/25544)
HLA-DRB1*0901	11.565 (34/294)	12.730 (28199/221520)	16.505 (4226/25605)
HLA-DRB1*1101	24.409 (62/254)	11.282 (25151/222928)	15.357 (3911/25467)
HLA-DRB1*1201	36.897 (107/290)	14.985 (33487/223464)	19.633 (5000/25467)
HLA-DRB1*1302	13.962 (37/265)	19.774 (44027/222646)	22.903 (5874/25647)
HLA-DRB1*1501	35.206 (94/267)	21.341 (47568/222893)	25.415 (6463/25430)
HLA-DRB3*0101	9.158 (25/273)	8.145 (18105/222274)	6.556 (1673/25517)
HLA-DRB3*0202	18.657 (50/268)	13.445 (29830/221859)	15.457 (3960/25620)
HLA-DRB4*0101	22.145 (64/289)	12.341 (27467/222568)	15.856 (4038/25467)
HLA-DRB5*0101	22.028 (63/286)	10.677 (23753/222464)	14.138 (3602/25478)

17 PRESENTATION OF TMH-DERIVED EPITOPES WHEN TWO AMINO ACIDS OVERLAP

In our experiment, we define a TMH-derived epitope as a peptide that overlaps with a TMH for at least one amino acid. One could argue that we should use a higher number of overlapping amino acids, so to make the epitopes more 'transmembrane helix-ey'. We chose not too, for two reason: (1) epitopes that overlap with a TMH for 1 AA already, cannot be processed by the proteasome in a known and conventional way (2) whatever number of overlapping amino acids we use, we expect the pattern to be the same. However, using only 1 AA gives the most TMH-derived epitopes and hence the highest statistical power.

To prove this point, we did exactly the same analysis as shown in Figure 1A, yet with defining a TMH-derived epitope as an epitope that overlaps with a TMH for at least 2 AAs, as shown in Figure S15. As these two figures look identical, we also added the counts as numbers, with Table S14 showing the same data as S5, except the former uses 2 AAs overlap. Likewise, Table S15 showing the same data as S7, except the former uses 2 AAs overlap.

ABBREVIATIONS

REFERENCES

Bianchi, F., Textor, J., and van den Bogaart, G. (2017). Transmembrane helices are an overlooked source of Major Histocompatibility Complex Class I epitopes. *Frontiers in immunology* 8, 1118

[Dataset] Bilderbeek, R. J. C. (2019a). netmhc2pan. `https://github.com/richelbilderbeek/netmhc2pan` [Accessed: 2019-03-08]

[Dataset] Bilderbeek, R. J. C. (2019b). tmhmm. `https://github.com/richelbilderbeek/tmhmm` [Accessed: 2019-03-08]

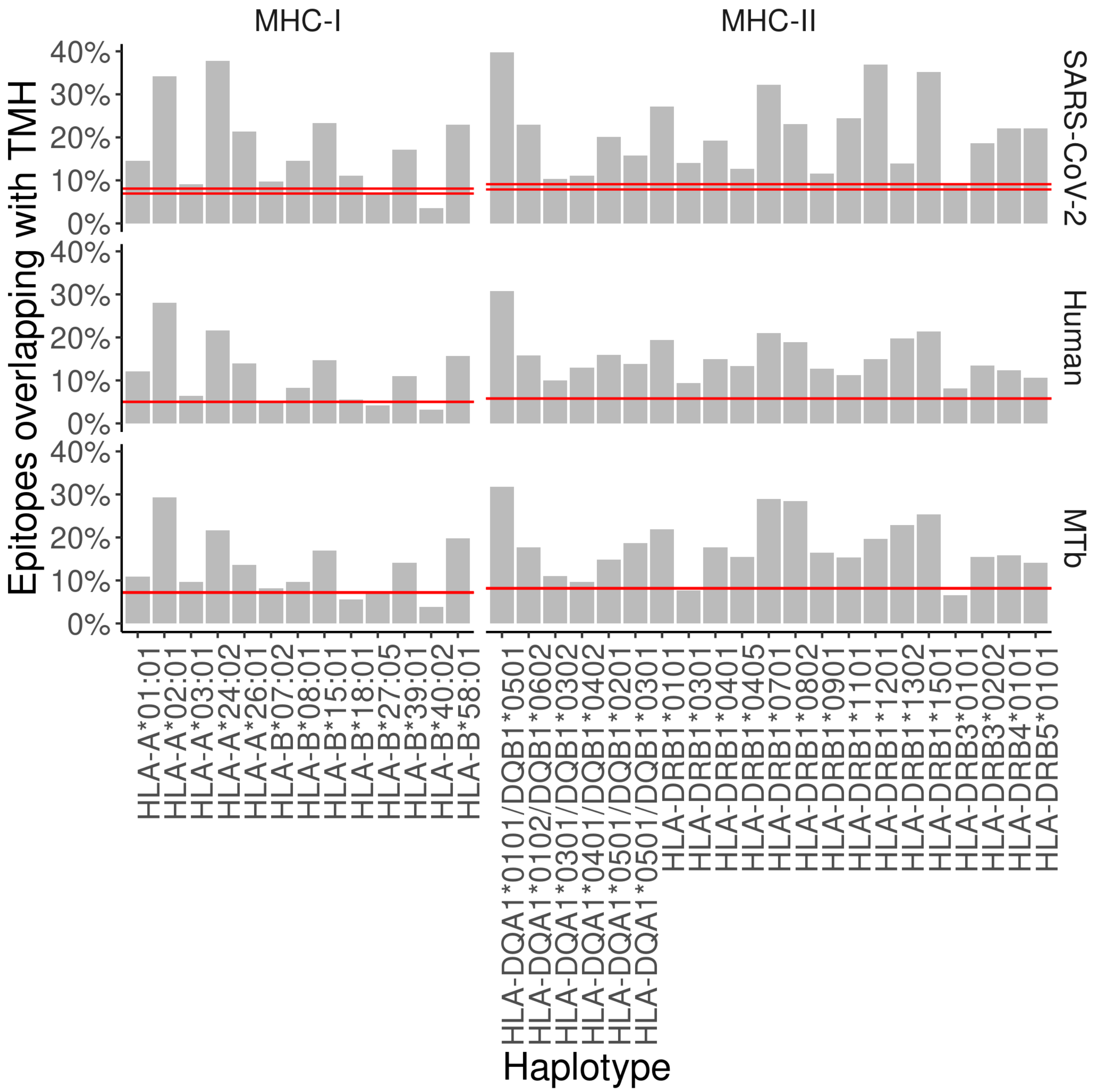

Figure S15. The percentage of epitopes for MHC-I and -II alleles that are predicted to overlap with TMHs (for at least two amino acids) for the proteomes of SARS-CoV-2 (top row), human (middle row) and *M. tuberculosis* (bottom row). The pair of dashed lines in each plot indicate the lower and upper bound of the 99% confidence interval. See supplementary Tables S14 and S15 for the exact TMH and epitope counts.

[Dataset] Bilderbeek, R. J. C. (2020a). bbbq. `https://github.com/richelbilderbeek/bbbq` [Accessed: 2020-09-02]

[Dataset] Bilderbeek, R. J. C. (2020b). pureseqtmr. `https://github.com/richelbilderbeek/pureseqtmr` [Accessed: 2020-05-19]

[Dataset] Bilderbeek, R. J. C. (2021a). iedbr. `https://github.com/richelbilderbeek/iedbr` [Accessed: 2021-11-09]

Table S15. Percentage of MHC-I 9-mers overlapping with TMH. Values in brackets show the number of binders that have at least two residues overlapping with a TMH (first value)as well as the number of binders (second value). percentage used: 2

haplotype	covid	human	myco
HLA-A*01:01	14.539 (41/282)	12.092 (27232/225209)	10.912 (2815/25797)
HLA-A*02:01	34.155 (97/284)	28.037 (63085/225003)	29.360 (7546/25702)
HLA-A*03:01	9.122 (27/296)	6.388 (14361/224796)	9.673 (2488/25721)
HLA-A*24:02	37.809 (107/283)	21.677 (48913/225648)	21.643 (5571/25741)
HLA-A*26:01	21.405 (64/299)	13.905 (31370/225598)	13.632 (3516/25793)
HLA-B*07:02	9.712 (27/278)	4.880 (10854/222429)	8.184 (2107/25744)
HLA-B*08:01	14.539 (41/282)	8.218 (18376/223616)	9.662 (2480/25667)
HLA-B*15:01	23.311 (69/296)	14.686 (33269/226542)	16.961 (4382/25835)
HLA-B*18:01	11.034 (32/290)	5.603 (12537/223745)	5.560 (1433/25773)
HLA-B*27:05	6.818 (18/264)	4.171 (9350/224178)	7.054 (1812/25688)
HLA-B*39:01	17.091 (47/275)	10.983 (24538/223419)	14.159 (3652/25793)
HLA-B*40:02	3.534 (10/283)	3.251 (7264/223408)	3.852 (991/25729)
HLA-B*58:01	22.939 (64/279)	15.627 (35022/224119)	19.793 (5095/25742)

Abbreviation	Full
ER	Endoplasmatic reticulum
ERAD	ER-associated degradation
HLA	Human leukocyte antigen
IEDB	Immune Epitope Database
LB	lipid body
MAP	Membrane-associated protein
MHC	Major histocompatibility complex
MVB	Multivesicular body
PLC	Peptide-loading complex
SNP	Single nucleotide polymorphism
TMH	Transmembrane helix
TMP	Transmembrane protein

[Dataset] Bilderbeek, R. J. C. (2021b). sprentrez. `https://github.com/richelbilderbeek/sprentrez` [Accessed: 2021-02-09]

Hunt, R., Sauna, Z. E., Ambudkar, S. V., Gottesman, M. M., and Kimchi-Sarfaty, C. (2009). Silent (synonymous) SNPs: should we care about them? *Single nucleotide polymorphisms* , 23–39

Karosiene, E., Lundegaard, C., Lund, O., and Nielsen, M. (2012). NetMHCcons: a consensus method for the major histocompatibility complex class I predictions. *Immunogenetics* 64, 177–186

Karosiene, E., Rasmussen, M., Blicher, T., Lund, O., Buus, S., and Nielsen, M. (2013). NetMHCIIpan-3.0, a common pan-specific MHC class II prediction method including all three human MHC class II isotypes, HLA-DR, HLA-DP and HLA-DQ. *Immunogenetics* 65, 711–724

Krogh, A., Larsson, B., Von Heijne, G., and Sonnhammer, E. L. (2001). Predicting transmembrane protein topology with a hidden Markov model: application to complete genomes. *Journal of molecular biology* 305, 567–580

Lundegaard, C., Lund, O., and Nielsen, M. (2011). Prediction of epitopes using neural network based methods. *Journal of immunological methods* 374, 26–34

Möller, S., Croning, M. D., and Apweiler, R. (2001). Evaluation of methods for the prediction of membrane spanning regions. *Bioinformatics* 17, 646–653

Musumeci, L., Arthur, J. W., Cheung, F. S., Hoque, A., Lippman, S., and Reichardt, J. K. (2010). Single nucleotide differences (SNDs) in the dbSNP database may lead to errors in genotyping and haplotyping studies. *Human mutation* 31, 67–73

Nielsen, M., Lundegaard, C., Blicher, T., Peters, B., Sette, A., Justesen, S., et al. (2008). Quantitative predictions of peptide binding to any HLA-DR molecule of known sequence: NetMHCIIpan. *PLoS computational biology* 4

Nielsen, M., Lundegaard, C., Worning, P., Hvid, C. S., Lamberth, K., Buus, S., et al. (2004). Improved prediction of MHC class I and class II epitopes using a novel Gibbs sampling approach. *Bioinformatics* 20, 1388–1397

Nielsen, M., Lundegaard, C., Worning, P., Lauemøller, S. L., Lamberth, K., Buus, S., et al. (2003). Reliable prediction of T-cell epitopes using neural networks with novel sequence representations. *Protein Science* 12, 1007–1017

Shao, X. M., Bhattacharya, R., Huang, J., Sivakumar, I. A., Tokheim, C., Zheng, L., et al. (2020). High-throughput prediction of MHC class I and II neoantigens with MHCnuggets. *Cancer Immunology Research* 8, 396–408

Vita, R., Mahajan, S., Overton, J. A., Dhanda, S. K., Martini, S., Cantrell, J. R., et al. (2019). The immune epitope database (iedb): 2018 update. *Nucleic acids research* 47, D339–D343

Wang, Q., Ni, C., Li, Z., Li, X., Han, R., Zhao, F., et al. (2019). PureseqTM: efficient and accurate prediction of transmembrane topology from amino acid sequence only. *bioRxiv* , 627307

Winter, D. J. (2017). rentrez: an R package for the NCBI eUtils API. *The R Journal* 9, 520–526
